# Symposia Abstracts

**DOI:** 10.1080/24740527.2019.1599263

**Published:** 2019-04-01

## Neuromodulation for Pain – Choosing the Right Modality for the Right Patient at the Right Time

Angela Mailis, Anuj Bhatia, and Amitabh Gulati

**CONTACT** Angela Mailis angela.mailis@uhn.ca

© 2019 The Author(s). Published with license by Taylor & Francis Group, LLC.

This is an Open Access article distributed under the terms of the Creative Commons Attribution License (http://creativecommons.org/licenses/by/4.0/), which permits unrestricted use, distribution, and reproduction in any medium, provided the original work is properly cited.

Neuromodulation including spinal cord (SCS) and peripheral nerve stimulation (PNS) is now increasingly available and it has a favorable benefit-to-risk profile with significant economic benefits for patients and the society. Traditionally, neuromodulation techniques are used to treat chronic neuropathic pain syndromes, but more recently, nociceptive pathologies have also been successfully treated. Offering neuromodulation early and to patients with appropriate indications are the keys to optimizing long-term outcomes. There is strong evidence to support benefits of SCS in peripheral neuropathic pain and its superiority over repeat surgery for patients with history of previous spine surgery. Paresthesia-based, tonic SCS (PB-SCS) has been extensively used to treat neuropathic pain in the limbs with or without axial pain with mean reduction in pain intensity of over 60%. However, PB-SCS suffers from limitations including attenuation of benefit with time and or problems with painful or unwanted paresthesias. Newer modes of SCS and recent advances in hardware for PNS have expanded the indications for neuromodulation and can improve the efficacy of neuromodulation in the pain population. This symposium will cover three key areas of current clinical and research interest – patient selection, mechanisms and outcomes of paresthesia-free SCS, and role of PNS in current pain management.

### Speaker 1 Abstract Title: Patient selection for neuromodualtion – who is likely to benefit and who will not

**Speaker 1**: Angela Mailis, MD MSc FRCPC, University Health Network, Pain and Wellness Centre, Department of Medicine, University of Toronto, Toronto, Ontario, Canada, Angela.Mailis@uhn.ca

**Speaker 1 Abstract**: Spinal cord stimulation can treat intense, unremitting pain effectively if offered for the correct indications with persistent pain post-laminectomy, complex regional pain syndrome (CRPS), and neuropathic pain secondary to diabetes and other etiologies being common indications. Patient selection is of paramount importance. Selecting patients for this intervention requires rigorous assessment consisting of a detailed history, a thorough examination, and appropriate investigations, including psychological assessment. Establishing reasonable patient expectations and clear communication between the referring and implanting healthcare teams is key to achieving high success rates. The pain physician should be involved in decision-making during and at the end of the trial process. Timely follow-ups after implantation are important to optimize outcomes of neuromodulation therapy. It is equally important to not offer neuromodulation to patients who are not psychologically ready or do not have appropriate indications with an incorrect diagnosis of CRPS being a common cause of failure of neuromodulation.

### Speaker 2 Abstract Title: New modes of spinal cord stimulation (SCS) – High Frequency, Burst, High Density, and DRG stimulation – mechanisms of action and outcomes

**Speaker 2**: Anuj Bhatia, MD FRCPC, University Health Network, Department of Anesthesia and Pain Medicine, University of Toronto, Toronto, Ontario, Canada, Anuj.Bhatia@uhn.ca

**Speaker 2 Abstract**: New modes of SCS promise to enhance success with SCS while avoiding many of the factors responsible for failure of conventional tonic SCS. The ability to stimulate analgesic pathways within the nervous system independent of stimulation of fibers responsible for paresthesias [paresthesia-free(PF) – SCS] can enhance patient acceptance of SCS. Further, new SCS modes have the potential to treat mixed nociceptiveneuropathic pain which is encountered commonly but difficult to treat. Novel SCS modes include – high frequency (including HF-10), high density (HDTM), and BurstTM. These modes share the property of delivering higher amounts of electric charge to the spinal cord, with frequencies in the range of 500 Hz to 10 kHz and stimulation amplitudes that are below the perception threshold, thus making these modes paresthesia-free. DRG stimulation relies on stimulating the dorsal root ganglion in patients with focal CRPS. In patients with failure of conventional or tonic paresthesia-based SCS to achieve analgesia, either at the time of SCS trial or loss of benefit over time despite initial success, increasing the stimulation frequency or changing the stimulation pattern to deliver PF-SCS could confer analgesic benefit despite the absence of paresthesias. Ongoing research into mechanisms of actions of these new modes and their outcomes as reported in recent multi-center, randomized controlled trials will be presented.

### Speaker 3 Abstract Title: It is not all about the spinal cord – peripheral nerve stimulation for pain

**Speaker 3**: Amitabh Gulati, MD, Memorial Sloan Kettering Cancer Centre, Department of Anesthesia and Pain Medicine, New York, New York, USA, AmitabhGulati@gmail.com

**Speaker 3 Abstract**: Peripheral nerve stimulation (PNS) has been used for treatment of neuropathic pain for more than 50 years but recent resurgence of interest and improved technology in this modality has expanded indications. Introduction of less-invasive implantation techniques, wide availability of ultrasound, and “battery-free” systems have increased use of this modality. PNS works well in both established indications, such as posttraumatic and postsurgical neuropathy, occipital neuralgia, and complex regional pain syndromes, and in relatively-new indications for neuromodulation, such as cancer pain, migraines, and persistent post-surgical pain syndromes. Future research and growing clinical experience will help in identifying the best candidates for PNS, choosing the best procedure and best hardware for each individual patient, and defining adequate expectations for patients and pain specialists.

**Learning Objective 1**: Attendees will be able to identify patients who can benefit from neuromodulation.

**Learning Objective 2**: Attendees will be able to understand mechanisms of new modes of spinal cord stimulation and the principles of evaluating outcomes of these modes in patients who trial these modes.

**Learning Objective 3**: Attendees will be able to recognize indications for the role of peripheral neuromodulation in patients with neuropathic pain.

## Formal Continuing Pain Education: How Can it Improve Patient Outcomes?

Judy Watt-Watson, Michelle Gagnon, and Thomas Hadjistavropoulos

**CONTACT** Thomas Hadjistavropoulos Thomas.Hadjistavropoulos@uregina.ca

© 2019 The Author(s). Published with license by Taylor & Francis Group, LLC.

This is an Open Access article distributed under the terms of the Creative Commons Attribution License (http://creativecommons.org/licenses/by/4.0/), which permits unrestricted use, distribution, and reproduction in any medium, provided the original work is properly cited.

**Symposium Chair**: Thomas Hadjistavropoulos, Ph.D., ABPP, FCAHS, Centre on Aging and Health, University of Regina, Regina, SK, Canada, Thomas.Hadjistavropoulos@uregina.ca, @URHealthPsycLab

**Symposium Abstract**: We will focus on continuing pain education training initiatives targeting health professionals working with children, vulnerable seniors with dementia as well as other adults. The need for formal continuing pain education cannot be understated. It is also clear that continuing pain education tends to increase participants’ knowledge about pain care. The extent to which such education leads to improved clinical practices and outcomes is less clear. We will review the literature in this area and introduce some new data with the aim of aim identifying elements that tend to increase the probability that continuing pain education will improve patient outcomes.

### Speaker 1 Abstract Title: Improving Pain Practices through Continuing Professional Development: Is Education Enough?

**Speaker 1**: Judy Watt-Watson, RN, MSc, PhD, Lawrence S. Bloomberg Faculty of Nursing, University of Toronto, Toronto, Ontario, Canada, j.watt.watson@utoronto.ca

**Speaker 1 Abstract**: Despite available resources including pain curricula and related foundational competencies, pain content at the prelicensure level is not a priority and is frequently minimal as evidenced by curriculum surveys worldwide. Moreover, pain competency is not a requirement for licensure and significant pain prevalence for people with acute and/or chronic pain continues to be documented. The Global Burden of Disease Survey concluded that “persistent pain is the most important, current future cause of morbidity & disability across the world & increasing” (Rice et al., 2013). While the focus on prelicensure education is a priority, ensuring that practitioners have evidence-based, current pain knowledge about best practices for managing pain is also essential. The public trusts health professionals to be knowledgeable and provide care that is safe, efficient, effective, timely, patient-centered, and equitable. Therefore, Continuing Professional Development can help practitioners to maintain, improve and broaden pain knowledge and skills related to their own needs and setting. There is no one approach for solving pain education challenges and evidence for a range of options will be examined. The application of the Pain Interprofessional Curriculum Design (PICD) Model for developing CPD initiatives in participants’ contexts will also be discussed.

### Speaker 2 Abstract Title: Increasing Knowledge of Evidence-Based Practice Among Health Professionals Working with Children and Parents

**Speaker 2**: Michelle Gagnon, Ph.D., Department of Psychology, University of Saskatchewan, Saskatoon, Saskatchewan, Canada, michelle.gagnon@usask.ca, @MicheGagnon

**Speaker 2 Abstract**: In spite of a surge in programs to improve dissemination and implementation of evidence-based practices in pediatric contexts, inadequacies in pain assessment and management remain. In addition to the need for health care provider education, parents are increasingly responsible for the pain management of their child, such as during vaccinations or post-operatively, which has led to a rise in educational and knowledge translation programs for parents. This presentation will review findings from research on programs developed to improve health care provider and parental knowledge about pediatric pain. First, evidence from a recently conducted systematic evaluation of health care provider programs will be reviewed. The significant variability in the quality and impact of education programs for health care providers will be discussed, along with identified barriers and facilitators to success. Second, given the increasing focus on translating research knowledge to end-users, such as parents, new data synthesizing parent-focused programs will be presented. Parent-targeted programs share distinct challenges and contributors to success in achieving outcomes than programs targeting health care providers. Although existing programs can result in changes to parental practices, improvements in pain-related domains are not achieved, and many programs do not lead to sustained change, nor are they available to parents. Lastly, integrating our findings from health care provider and parent education, recommendations for future program development will be discussed, highlighting the need for innovative approaches and reduction of research waste.

### Speaker 3 Abstract Title: Continuing pain education in long-term care: Does it improve patient outcomes?

**Speaker 3**: Thomas Hadjistavropoulos, Ph.D., ABPP, FCAHS, Centre on Aging and Health, University of Regina, Regina, SK, Canada, Thomas.Hadjistavropoulos@uregina.ca, @URHealthPsycLab

**Speaker 3 Abstract**: Research examining long-term care (LTC) staff knowledge has documented deficits about key aspects of pain/pain care. Findings such as these underscore the importance of continuing pain education (CPE) in LTC facilities. A series of studies conducted both by our group and by others, have documented increases in knowledge when CPE is offered through a variety of formats (e.g., workshops, video presentation, interactive internet-based training). Our data show that, when such training is accompanied by a systematic implementation plan supported by management, improvements in clinical practice quality indicators occur (e.g., increased frequency of pain assessments using a standardized tool). At the same time, our evidence suggests that CPE, that is not accompanied by an implementation plan supported by management, is unlikely to lead to widespread practice changes in an LTC environment. Given that CPE can be costly for both staff and LTC homes, it is important that it be offered in a manner that will lead to improvements in patient care. Recommendations, based both on research evidence and on continuing education/implementation experience, will be presented to facilitate the translation of continuing education knowledge into widespread, irreversible and positive change in clinical practice.

**Learning Objective 1**: To familiarize participants with types and outcomes of formal continuing pain education initiatives involving health professionals who work with people of all ages.

**Learning Objective 2**: To examine the relevance of the Pain Interprofessional Curriculum Design Model to continuing professional pain education contexts.

**Learning Objective 3**: To familiarize participants with factors that tend to increase the probability that continuing professional education will lead to improvements in patient outcomes.

## Is Enhanced Pain Facilitation And/Or Impairment in the Efficacy of the Endogenous Inhibitory Pain System an Important Contributing Factor in Chronic Pain?

Catherine E. Ferland, Guillaume Leonard, and Karen D. Davis 0000-0003-1879-0090

**CONTACT** Catherine E. Ferland catherine.ferland@mcgill.ca

© 2019 The Author(s). Published with license by Taylor & Francis Group, LLC.

This is an Open Access article distributed under the terms of the Creative Commons Attribution License (http://creativecommons.org/licenses/by/4.0/), which permits unrestricted use, distribution, and reproduction in any medium, provided the original work is properly cited.

**Symposium Chair:** Catherine E. Ferland, PhD, McGill University, Anesthesia, Montreal, Quebec, Canada, Catherine.ferland@mcgill.ca

**Symposium Abstract:** Several factors may lead to poor pain management and consequently to the development of chronic pain, recognized as an expensive and debilitating public health issue. One abnormality associated with chronic pain is enhanced pain facilitation that can involve excitatory mechanisms such as central sensitization. A second abnormality associated with chronic pain is deficits in the endogenous mechanisms of pain control; possibly due to reduced efficacy of the endogenous inhibitory efferent pathways. Patients with sub-optimal function of this system are more likely to have poor pain control. This Symposium will examine brain-behaviour links related to pain facilitation (reflected by temporal summation of pain) and pain modulation (as reflected by conditioned pain modulation) in experimental and clinical studies.

### Speaker 1 Abstract Title: Endogenous pain modulation in youth with musculosketal pain: psychophysical findings and clinical implications

**Speaker 1**: Catherine E. Ferland, PhD, McGill University, Anesthesia, Montreal, Quebec, Canada, Catherine.ferland@mcgill.ca

**Speaker 1 Abstract**: Although several studies have reported altered pain sensitivity in different chronic pain conditions, there is sparse report on the interplay between peripheral input and central pain modulation through facilitatory and inhibitory pain mechanisms in paediatric patients with musculoskeletal (MSK) pain. Over the last year, we have been investigating pain processes in patients who presented at the spine clinics for long-term back pain. Conducted at the Shriners Hospital, we evaluated the endogenous pain modulation of 102 patients with scoliosis that reported presence of chronic pain. Findings suggest that despite the same pathology leading to a chronic pain state, different central pain modulating responses are observed among patients, suggesting that the pain management intervention should not be homogenous among all patients, an ill practice that still prevails today. Psychophysical findings throughout the peri-operative period of a cohort of adolescents who underwent major orthopaedic surgery will also be presented to discuss the mechanisms driving the development of poor postoperative pain control and individual predisposition to progress towards a chronic pain state. Based on preliminary findings, treatment modalities appear to be ineffective in one out of five. We will conclude with a discussion of the clinical implications of these findings, and how the standardized best practice for peri-operative pain management could be personalized for children undergoing similar surgical procedures but with different pain experience.

### Speaker 2 Abstract Title: Endogenous pain modulation in the elderly: psychophysical findings and clinical implications

**Speaker 2**: Guillaume Leonard, Pht, Ph.D., Research Center on Aging – Université de Sherbrooke, École de réadaptation, Sherbrooke, Quebec, Canada, guillaume.leonard@usherbrooke.ca

**Speaker 2 Abstract**: Chronic pain is well known to be particularly prevalent in the elderly population. The changes in endogenous pain modulation systems observed with aging have been proposed as a possible underlying factor of this increased prevalence. As such, a better understanding of these modulating systems and their influence on the occurrence and severity of pain in elderly patients could guide clinical decision-making, and pave the way for new treatment avenues. We will explore the interactions between ageing, endogenous pain-modulating mechanisms, and chronic pain, beginning with a review of seminal work on the subject. We will then present novel data on the correlation between age and facilitatory/inhibitory pain responses (temporal summation and conditioned pain modulation, respectively) obtained by our research team in a study conducted on 103 healthy participants aged 18 to 79 years old. We will also discuss current evidence on the relationship between pain symptoms (e.g. pain intensity, interference with physical function) and endogenous modulating mechanisms in chronic pain patients, before offering preliminary results obtained by our team on this relationship as seen in patients suffering from knee osteoarthritis. We will conclude with a discussion of the clinical implications of these findings, and consider how they can be integrated in a clinical setting to provide personalized and adapted health care to the ageing population.

### Speaker 3 Abstract Title: Contribution of bottom-up, top-down, and intrinsic activity in the dynamic pain connectome reflect individual pain sensitivity and chronic pain treatment response

**Speaker 3**: Karen D. Davis, PhD, Krembil Research Institute, Division of Brain, Imaging and Behaviour – System Neuroscience, Toronto, ON, karen.davis@uhnresearch.ca, @kren27

**Speaker 3 Abstract**: The nociceptive spectrum framework proposed by Yarnitsky indicates that individuals fall on a behavioural continuum from pro- to anti-nociceptive as characterized by their pain sensitivity (threshold, temporal summation of pain, conditioned pain modulation). Our lab has conducted brain imaging studies to examine the brain mechanisms underlying this behavioural designation. Thus, this talk will first present data showing that individual brains exhibit features that lie on a spectrum from pro- to anti-nociceptive as revealed through 1) studies of the functional connectivity of the ascending (bottom – up) pathway and the descending (top-down) pathway (Cheng et al., J. Neurosci 2015), and 2) of intrinsic activity in the dynamic pain connectome that show sex-differences (Rogachov et al., Pain 2016). These data in healthy individuals indicate that it is the balance of strength of the ascending vs descending pathways that dictate pro- vs anti-nociceptivity. Based on this concept, I will then discuss our recent study that found that the analgesic effect of ketamine in patients with neuropathic pain could be predicted by the strength of these 2 systems reflected by temporal summation of pain and dynamic functional connectivity measures (Bosma et al., Anesthesiology 2018).

**Learning Objective 1**: To recognize the differences in the endogenous pain modulation among paediatric cohorts with pain conditions and at risk of poor acute and chronic pain management.

**Learning Objective 2**: To explore the role played by endogenous pain modulation in the occurrence of pain conditions and severity of pain symptoms in elderly individuals.

**Learning Objective 3**: To understand how brain imaging and psychophysics can be used to link brain mechanisms to the spectrum of behavioural pain sensitivity and to predict chronic pain treatment efficacy.

## Pain in Autoimmune Disease

Bradley Kerr, Nader Ghasemlou, and Ji Zhang

**CONTACT** Bradley Kerr bjkerr@ualberta.ca

© 2019 The Author(s). Published with license by Taylor & Francis Group, LLC.

This is an Open Access article distributed under the terms of the Creative Commons Attribution License (http://creativecommons.org/licenses/by/4.0/), which permits unrestricted use, distribution, and reproduction in any medium, provided the original work is properly cited.

**Symposium Abstract**: Multiple sclerosis (MS) and Guillain Barre Syndrome (GBS) are the two most frequently observed forms of autoimmune neuropathy in clinics. Often masked by muscle weakness and progressive paralysis, pain, although invisible, occurs often in these patients and is one of the most long-lasting sequelae of autoimmune neuropathy. However, the pathophysiology of pain in autoimmune disease is poorly understood. In this workshop, we will 1) discuss the animal model commonly used to study MS and examine new methodologies that allow us to analyze changes in sensory function while removing many of the confounds of motor impairment; 2) discuss the most recent findings on neuronal mechanisms of pain in MS highlighting the role of peripheral sensory ganglia in this process; 3) demonstrate the evidence of viral infection and injury triggered GBS like symptoms in mice and discuss the key role of CD8 T cell-macrophage interaction in autoimmune peripheral neuropathy-associated chronic pain.

### Speaker 1 Abstract Title: The role of the peripheral nervous system in central neuropathic pain: Changes in primary sensory neurons in an animal model of CNS autoimmune demyelination

**Speaker 1: Bradley Kerr**, PhD, University of Alberta, Department of Anesthesiology and Pain Medicine, Alberta, Canada. bjkerr@ualberta.ca. @BradleyKerr20

**Speaker 1 Abstract**: MS is an autoimmune inflammatory and neurodegenerative disease that is associated with significant demyelination of axonal tracts in the brain and spinal cord. Demyelinating plaques underlie the pathological signs of weakness and paralysis that is most commonly associated with the disease. However, a significant proportion of patients with MS also develop sensory disturbances including neuropathic pain in the distal limbs and/or trigeminal neuralgia. In this talk I will present recent data implicating the peripheral nervous system, specifically the sensory neurons of the DRG and TG, as key drivers of neuropathic pain in this disease state. I will discuss recent insights into the maladaptive neuronal plasticity that occurs in the primary sensory neurons even in the absence of overt demyelination. I will discuss how this plasticity affects pain sensitivity in a specific mouse model used to study MS. Sex differences in these responses will also be discussed. After attending this talk learners will have a greater appreciation of the contribution made by the peripheral nervous system towards generating central neuropathic pain states.

### Speaker 2 Abstract Title: New approaches to modeling pain in MS

**Speaker 2: Nader Ghasemlou**, PhD, Queen’s University, Department of Anesthesiology and Pain Medicine, Kingston, Ontario, Canada. Nader.ghasemlou@queensu.ca

**Speaker 2 Abstract**: Chronic pain is a highly prevalent concomitant disorder of multiple sclerosis (MS). A commonly-used model of MS, experimental autoimmune encephalomyelitis (EAE), typically presents with hindlimb paralysis, neuroinflammation and neurodegeneration. However, this paralysis hinders the use of pain behavior tests, which rely heavily on the mobility of the animal. We therefore sought to adapt the classic actively-induced EAE model in order to optimize its pain phenotype – in essence dissociating motor deficits from sensory phenotypes. EAE was induced with 50μg MOG_35-55_ emulsified with 1mg/ml CFA and injected at two sites on the lower back subcutaneously, with 100, 200, 400, or 600ng of pertussis toxin (PTX) injected intraperitoneally. All mice were assessed for mechanical, cold and thermal sensitivity with the von Frey, acetone, and Hargreaves tests, respectively, over a period of 28 days. Spinal cord tissue was collected at 14 and 28 days post-injection to assess demyelination and neuroinflammation using immunohistochemistry and flow cytometry. results indicate that a lower severity of EAE disease is optimal for studying pain behaviours while still presenting with the pathology of EAE. Mice injected with 100ng PTX show significant mechanical and cold hypersensitivity. Using myelin staining, immunostaining, and flow cytometry, we have shown that despite the low clinical score produced with 100ng PTX, these mice nonetheless present with demyelination and neuroinflammation at similar levels to mice with more severe clinical symptoms. By using this modified EAE model, we will be able to better study the mechanisms underlying pain in autoimmune diseases, potentially leading to the development of new therapeutic targets to more effectively treat pain.

### Speaker 3 Abstract Title: The essentials of CD8 T cell-macrophage interaction in autoimmune peripheral neuropathy and associated chronic pain

**Speaker 3: Ji Zhang**, (MD, PhD), McGill University, The Alan Edwards Centre for Research on Pain, Montreal, Quebec, Canada, Ji.Zhang@mcgill.ca

**Speaker 3 Abstract**: Autoimmune peripheral neuropathy (APN) such as Guillain Barre Syndrome (GBS) and chronic inflammatory demyelinating polyneuropathy (CIDP) is a debilitating illness and can sometimes be life threatening. The molecular and cellular mechanisms remain elusive but exposure to environmental factors including viral/bacterial infection and injury is highly associated with disease incidence. Although the most striking and alarming feature in GBS patients are progressive weakness and paralysis, pain occurs frequently, often starting before onset of weakness and persisting for years. I will present our recent study using transgenic animals and viral infection to highlight some essentials in APN pathogenesis; including 1) how viral infection is linked to APN; 2) in what circumstances, an injury can trigger APN; 3) what the critical roles of CD8 T cells and nerve macrophages are in mediating APN-associated pain. Our findings reveal that indeed, the synergism between CD8^+^ T cells and co-stimulation competent nerve macrophages is crucial in inducing autoimmune-mediated peripheral neuropathy. The identification of decisive molecular/cellular players connecting environmental triggers and the occurrence of APN provides opportunities to prevent disease onset, reduce relapses and develop new therapeutic strategies.

**Learning Objective 1**: After this symposium, the learner will become familiar with novel animal models that model the pathophysiology of different autoimmune diseases.

**Learning Objective 2**: After this symposium, the learner will understand how the peripheral nervous system reacts and impacts on sensory function in disease states primarily affecting the CNS.

**Learning Objective 3**: After this symposium, the learner will gain insight into novel, immunological mechanisms that lead to pain in autoimmune peripheral neuropathy.

## Pain after Traumatic Brain Injury: A Clinical and Molecular Perspective Towards Better Management and Prevention

Gilles Lavigne, Céline Gélinas, Caroline Arbour, and Samar Khoury

**CONTACT** Gilles Lavigne gilles.lavigne@umontreal.ca

© 2019 The Author(s). Published with license by Taylor & Francis Group, LLC.

This is an Open Access article distributed under the terms of the Creative Commons Attribution License (http://creativecommons.org/licenses/by/4.0/), which permits unrestricted use, distribution, and reproduction in any medium, provided the original work is properly cited.

**Symposium Chair**: Gilles Lavigne, DMD, PhD, Université de Montréal, Hôpital du Sacré-Coeur de Montréal, Montréal, Qc, Canada, gilles.lavigne@umontreal.ca

**Symposium Abstract**: Pain relief is a challenge in the context of traumatic brain injury (TBI) as many patients are temporarily unable to self-report. This is concerning because chronic pain is one of the most enduring sequelae of TBI and poorly managed pain in the acute phase of recovery could play a role in its development. Prevention of pain chronicity in this patient group starts with the use of validated tools to detect signs of unalleviated pain. A better understanding of the determinants associated to the emergence and maintenance of pain after TBI could also help clinicians identify at risk patients during the early stages of recovery. Ultimately, digging into the genetic profile of TBI individuals with chronic pain offers a new opportunity to match affected patients to suitable treatments. This symposium brings together clinicians and basic scientists to give an overview of the recent breakthroughs in our understanding of risk factors and preventive strategies for the alleviation of pain after TBI. After providing a brief introduction on the challenges surrounding pain assessment after TBI, Céline Gélinas will discuss her latest work regarding the adaptation of a behavioral pain scale for critically ill brain trauma patients. Caroline Arbour will describe the early clinical profile of TBI patients with persistent pain and investigate the possible underlying mechanisms. The session will conclude with a presentation from Samar Khoury, who will wrap-up the session and present emerging evidence supporting the plus value of genetic profiling to understand and treat chronic pain after TBI.

### Speaker 1 Abstract Title: Pain assessment in critically ill brain-injured patients: Filling a gap into practice

**Speaker 1**: Céline Gélinas, RN, PhD, McGill University, Centre for Nursing Research and Lady Davis Institute of the Jewish General Hospital, Montréal, Qc, Canada, celine.gelinas@mcgill.ca

**Speaker 1 Abstract**: Acute pain is a risk factor of chronic pain development. Brain-injured patients may experience moderate to severe pain in the critical phase of their recovery often spent in the intensive care unit (ICU). During this period, pain assessment is challenging as patients may be unable to self-report due to their brain injury and critical care condition. In such situations, behavioral pain scales are alternative measures. Previous studies and our recent work (n = 147) have shown that brain-injured ICU patients express specific pain behaviors. For instance, grimace and muscle rigidity were not frequently observed, and tearing and face flushing were newly described. These findings supported the need to adapt the content of existing scales to make them more suited to this vulnerable population. During this presentation, I will describe the steps undertaken in the adaptation and validation of a behavioral pain scale (i.e., Critical-Care Pain Observation Tool of CPOT) for use in critically ill brain-injured patients. First, I will present findings about the relevance of behaviors for pain assessment in this patient population from the perspective of ICU clinicians (n = 77) from four neuroscience ICUs in Canada and United States. Second, I will discuss the modifications made to the scale which is called CPOT-Neuro. Third, I will present findings from the validation of the CPOT-Neuro in 226 critically ill brain-injured patients from these four ICUs and its ability to detect pain. Finally, I will describe the nurses’ evaluations of the CPOT-Neuro use and key elements to consider for implementation.

### Speaker 2 Abstract Title: Early identification of patients at risk of chronic pain after TBI: How thinking outside the box could get us a long way

**Speaker 2**: Caroline Arbour, RN, PhD, Université de Montréal, Hôpital du Sacré-Coeur de Montréal, Montréal, Qc, Canada, caroline.arbour@umontreal.ca

**Speaker 2 Abstract**: Despite colossal efforts from clinicians to optimize recovery following major trauma, chronic pain remains the most common consequence of moderate-to-severe traumatic brain injury (TBI). Intriguingly, even with similar injury, only a portion of individuals will develop chronic pain after TBI (between 32% and 91% depending on the study). Until recently, the clinical profile of patients more likely to develop chronic pain after TBI was largely unknown. We were the first to demonstrate in a sample of N = 41 young adults that the co-occurrence of non-displaced spine fracture almost doubled the risk of developing chronic pain. Our most recent work with a large cohort of TBI survivors (N = 181) combining measures of pupillometry, heart rate variability, somatosensory evoked potentials and dosage of inflammatory markers shows that a cascade of autonomic and neurophysiological changes associated with persistent pain begin in the first three days following TBI. These screening elements could easily be implemented in any trauma units. Identifying TBI patients at greater risk of chronic pain within the first few days of hospital admission would support the deployment of analgesic adjustment strategies and interprofessional follow-up. In the near future, such information could be used for the targeted assignment of TBI patients to complementary therapies for pain relief. Indeed, the validation of several non-pharmacological interventions to optimize the management of refractory pain is imminent including promising neuromodulation therapies. Considering these interventions will require an investment of time from clinicians, identification of patients to whom they will be most beneficial will promote more efficient administration.

### Speaker 3 Abstract Title: Using genetics to predict chronic pain in mild traumatic brain injury

**Speaker 3**: Samar Khoury, PhD, McGill University, Montréal, Qc, Canada, samar.khoury2@mail.mcgill.ca

**Speaker 3 Abstract**: Historically, mild traumatic brain injury (mTBI) was considered a concussion that would heal in a relatively short time. More recently, mTBI represents a “silent epidemic” because it often leaves patients with post-concussion symptoms such as headaches, mood disturbances, deteriorated quality of life, sleepiness, and chronic pain. Genetic factors have been proposed to explain part of the variability in individual outcomes following mTBI. Even though many advances in genetics have been made in deciphering neurological and cognitive dysfunctions following mTBI, namely the roles of *APOE4, GFAP, S100beta*, and *DRD2*, very few studies have looked at the genes that predispose mTBI patients to develop chronic pain. First, I will give an overview of the known genes contributing to pain following mild traumatic injury. Then, I will present a recently published study in a cohort of 92 patients with mTBI that were followed up for one year and that were characterized for a plethora of pain phenotypes. In this study, we showed that chronic pain following mTBI is modulated by a multiple locus effect in the brain derived neurotrophic factor (*BDNF*) gene through the expression of its antisense. At last, I will discuss the new advances in the use of “omics” in the study of pain following traumatic brain injury.

**Learning Objective 1**: To share recent advances in pain assessment in the critical phase of TBI recovery

**Learning Objective 2**: Gain new insight into the clinical profile of TBI individuals who are at risk of transitioning from acute to chronic pain

**Learning Objective 3**: Project how genetics can be used to understand and treat chronic pain after TBI

## Advances in Magnetic Resonance Imaging of Human Spinal Cord: Challenges and Opportunities for Pain Researchers and Clinicians

Christian Buchel, Ali Khatibi 0000-0003-0679-0499, and Robert L. Barry

**CONTACT** Ali Khatibi ali.khatibi@gmail.com

© 2019 The Author(s). Published with license by Taylor & Francis Group, LLC.

This is an Open Access article distributed under the terms of the Creative Commons Attribution License (http://creativecommons.org/licenses/by/4.0/), which permits unrestricted use, distribution, and reproduction in any medium, provided the original work is properly cited.

**Symposium Chair**: Ali Khatibi, Ph.D., Department of Neurology and Neurosurgery, McGill University, Montreal, Quebec, Canada, ali.khatibi@gmail.com, @Aliikhatibi

**Symposium Abstract**: The spinal cord has long been known to be an important part of the central nervous system especially when it comes to the study of pain processing and its modulation. The spinal cord has received considerable attention in animal model studies, but some limitations (e.g., small diameter, physiological noise, high diversity in the shape) have hindered studying the spinal cord in living humans. Recent advances in magnetic resonance imaging (e.g., development of new tools, improvement of sequences and machines) have allowed researchers to study the structure and the function of the spinal cord *in vivo*. This symposium will present the state-of-the-art in structural and functional imaging of the human spinal cord, and describe the existing opportunities and challenges in this field. We will present specific examples of neuroimaging studies that focus on the role of the spinal cord in the processing and modulation of pain in humans. We will deliver guidelines and suggestions for future experimental and clinical studies interested in imaging the human spinal cord.

### Speaker 1 Abstract Title: Combined fMRI of the brain and the spinal cord in pain research

**Speaker 1**: Christian Buchel, M.D., Department of Systems Neuroscience, University Medical Center Hamburg-Eppendorf, Hamburg, Germany, buechel@uke.de, @C_Buchel

**Speaker 1 Abstract**: The dynamic interaction between ascending spino-cortical nociceptive signalling and the descending cortical control involving brainstem regions such as the periaqueductal gray matter (PAG) plays a critical role in acute and chronic pain. In the last decade, fMRI of the spinal cord made it possible to directly observe the integration of descending and ascending nociceptive processes at the dorsal horn. However, to noninvasively investigate the processing of nociceptive stimuli along the whole neuraxis, a combined spino-cortical acquisition scheme was required. Consequently, we developed a protocol with two separate slice stacks (brain and spinal cord), individually adapted resolutions and parameter settings that are dynamically updated to the optimized settings for the respective region. This protocol allowed to assess fMRI signals in the spinal cord and in the brain within one measurement. Since early investigations of the spinal cord-brainstem (PAG) axis in acute pain, we have also characterized modulation of pain through expectation (nocebo) and described this process across the brain and the spinal cord using the combined protocol. In this presentation, we will present novel aspects of this technique addressing the speed of acquisition, signal dropouts, image resolution and exemplify these methodological advances in several example applications.

### Speaker 2 Abstract Title: Understanding and modelling physiological noise in functional imaging of the human spinal cord

**Speaker 2**: Ali Khatibi, Ph.D., Department of Neurology and Neurosurgery, McGill University, Montreal, Quebec, Canada, ali.khatibi@gmail.com, @Aliikhatibi

**Speaker 2 Abstract**: Acquisition of BOLD signal from the human spinal cord has long been appealing for pain researchers but differentiating the signal from the noises like those as the result of physiological fluctuations made it very difficult to be conclusive. Both the respiration and the heart pulsation have proven to be major sources of variation in the noise in the acquired BOLD signal. Important for experimental pain studies, painful stimulation has shown to be associated with changes in the respiration and heart rate in subjects and thus may influence the acquired signal in the spinal cord and increases the chance for both type-I&II errors. We investigated the effect of different methods of physiological noise removal on the output of fMRI analysis in the human spinal cord and compared the results. We will present results from different studies focused on the functional imaging of the cervicothoracic spinal cord and the thoracolumbar spinal cord, in rest, during painful stimulation and during motor practice.

### Speaker 3 Abstract Title: Magnetic resonance imaging (MRI) of the human spinal cord at 7 Tesla

**Speaker 3**: Robert L. Barry, Ph.D., Athinoula A. Martinos Center for Biomedical Imaging, Department of Radiology, Massachusetts General Hospital, Harvard Medical School, Boston, MA, U.S.A., robert.barry@mgh.harvard.edu, @Researcher_Rob

**Speaker 3 Abstract**: Magnetic resonance imaging (MRI) of the human spinal cord at 7 Tesla has been demonstrated by a handful of research sites worldwide, and the spinal cord remains one of the areas in which higher fields and resolution could have high impact. The small diameter of the cord necessitates high spatial resolution to minimize partial volume effects between gray and white matter, and so MRI of the cord can greatly benefit from increased signal-to-noise ratio and contrasts at 7 Tesla. Spinal cord functional MRI (fMRI) has also been demonstrated at 7 Tesla, and offers higher sensitivity to blood oxygenation level dependent (BOLD) signal changes at higher spatial resolutions. However, technical challenges to successful spinal cord MRI and fMRI at 7 Tesla include radiofrequency (B1) nonuniformities and a general lack of optimized radiofrequency coils, amplified physiological noise, and an absence of methods for robust main field (B0) shimming along the cord. Numerous solutions to address these challenges have been and are continuing to be explored, including novel approaches for signal excitation and acquisition, dynamic shimming, and specialized shim coils.

**Learning Objective 1**: Understanding the interplay of the ascending and descending nociceptive system from the dorsal horn to the cortex

**Learning Objective 2**: Understanding the importance of modelling physiological noise in functional imaging of the human spinal cord

**Learning Objective 3**: Exploring the challenges and opportunities of spinal cord imaging at ultra-high magnetic fields

## Exploring Pain as a Multidimensional Experience: The Essential Role of Qualitative Research

Judy Watt-Watson, Fiona Webster, Craig Dale, and Nida Mustafa

**CONTACT** Judy Watt-Watson j.watt.watson@utoronto.ca

© 2019 The Author(s). Published with license by Taylor & Francis Group, LLC.

This is an Open Access article distributed under the terms of the Creative Commons Attribution License (http://creativecommons.org/licenses/by/4.0/), which permits unrestricted use, distribution, and reproduction in any medium, provided the original work is properly cited.

**Symposium Chair**: Judy Watt-Watson, RN, MSc, PhD, University of Toronto, Lawrence S. Bloomberg Faculty of Nursing, Toronto, Ontario, Canada, j.watt.watson@utoronto.ca, @jwattwatson1

**Symposium Abstract**: Pain is defined as a multidimensional experience – a highly subjective phenomenon resulting from the interaction of physical, biochemical, physiological, cognitive, emotional, behavioral, and sociocultural factors. Pain is complex, context-sensitive, and often resistant to objective measurement. Research that focuses upon the subjective nature of pain can contribute to understanding of the manifold ways in which pain is experienced in clinical and nonclinical contexts. This is important as patients and clinicians continue to identify deficiencies in all aspects of acute and chronic pain management despite growing biomedical understandings of its causes and consequences. Qualitative methods of engaging patients and clinicians in pain science are strongly recommended by the Canadian Institutes of Health Research (CIHR) and the International Association of the Study of Pain (IASP). Patient and caregiver experiences are now identified as key pieces of evidence to inform clinical pain services, health professional training, experimental interventions, and topics for research investment. In this presentation, we offer examples of qualitative explorations that have changed our understanding of pain, offer insight into the potential facilitators and barriers to good pain management, and generate critical directions for future research.

### Speaker 1 Abstract Title: Narratives from learners about treating patients with chronic pain

**Speaker 1**: Fiona Webster, PhD, University of Toronto, Institute of Health Policy Management and Evaluation (IHPME), Toronto, Ontario, Canada, Fiona.Webster@utoronto.ca @FionaWebster1

**Speaker 1 Abstract**: Chronic pain is widely acknowledged to be a growing health problem globally, and a growing health care concern in Canada. Physician empathy is an important component of care for patients with chronic pain. Yet while evidence suggests that student empathy is shaped by medical education, empathy is rarely explicitly taught. Moreover, studies surveying medical students’ attitudes towards chronic non-cancer pain patients suggest they are mainly negative, and that medical trainees become less empathetic and less patient-centered over the course of their training. Using a constructivist approach, our qualitative study explored investigated differences in the attitudes and beliefs of medical trainees towards caring for patients with chronic pain at different stages of training, with an emphasis placed on trainees’ reflections on the role of empathy in providing care for these patients. Drawing on interview data with medical students and residents at different stages of training, this presentation draws on Dorothy Smith’s broad concept of work in order to understand medical trainees’ understanding of empathy and its relevance for medical training.

### Speaker 2 Abstract Title: Making pain visible through video and photo-elicitation

**Speaker 2**: Craig Dale, RN PhD, University of Toronto, Lawrence S. Bloomberg Faculty of Nursing, Toronto, Ontario, Canada, craig.dale@utoronto.ca @craig_dale1

**Speaker 2 Abstract**: Cumulative research identifies approximately 70% of intensive care unit (ICU) patients experience unrecognized or undertreated pain. Pain during critical illness has serious physical and psychological effects, and can profoundly impair patient recovery, discharge, and rehabilitation. A frequently underestimated source of pain is routine medical and nursing procedures. Such pain may go unrecognized due to patient inability to communicate (e.g., secondary to advanced airways, reduced level of consciousness) and a busy ICU context. Video and photography are established qualitative research tools for exploring and analyzing real-world care delivery problems. In this presentation, we describe data from an ICU oral care study which employed video and photographic elicitation – a method which integrates visual data from actual patient encounters during interviews to explore interprofessional perceptions and recommendations inclusive of pain. We draw on critical social theory to consider the difficulty of discussing failures in pain recognition and the potential for visual techniques to neutralize these tensions – so that clinicians can be creatively engaged in seeing and resolving the under-recognition of pain and its negative outcomes.

### Speaker 3 Abstract Title: The Influence of Context: Exploring immigrant Indian women’s lived-experiences of chronic pain in Canada

**Speaker 3**: Nida Mustafa, BSc., MHSc., PhD Candidate, University of Toronto, Dalla Lana School of Public Health, Toronto, Ontario, Canada, nida.mustafa@utoronto.ca

**Speaker 3 Abstract**: As a growing public health concern, chronic pain affects approximately 1.5 million people in Canada, and is associated with decreased quality of life. Chronic pain is a growing issue among the rising immigrant population, as immigrant groups report higher pain intensity than non-immigrants. In 2011, the Indian population became the largest visible minority group, and continues to be the fastest growing. While the prevalence of chronic pain among Canadian-Indians is unknown, generally chronic pain has a higher prevalence among Indian women than men, with women reporting more severe and intense pain. An understanding of how pain is experienced by this particular group is therefore important for providing culturally-sensitive care. In this presentation, I discuss findings from a community-based study exploring Canadian-Indian women’s lived-experiences of chronic pain. Women participated in an interview and photovoice activity, in which pain experiences were captured using photographs. Analyses from interview and photograph data show women’s pain narratives are shaped by a myriad of socio-cultural factors, the most prominent being their gendered role as Indian women. Participants identified a stressful ‘dual-role’ after immigration, in which they are expected to balance traditional household duties and responsibilities of family, alongside employment outside the home. Many women revealed that their pain began, or significantly intensified, after immigrating to Canada. This qualitative research not only shows the significance of understanding the socio-cultural dimension of the pain experience, but also identifies factors within this context which may place particular groups of women at more risk of having lives filled with pain.

**Learning Objective 1**: To identify qualitative approaches to the exploration of pain across clinical and community settings.

**Learning Objective 2**: To describe social theory as a powerful means of seeing and articulating pain as a multidimensional phenomenon.

**Learning Objective 3**: To consider how qualitative evidence can inform clinical pain services, health professional training, experimental interventions, and topics for research investment.

## Cognitive Modulation of Pain: An Innovative Multidisciplinary, Multi-Species Approach

Mathieu Roy, Zoha Deldar, and Loren Martin

**CONTACT** Loren J. Martin lj.martin@utoronto.ca

© 2019 The Author(s). Published with license by Taylor & Francis Group, LLC.

This is an Open Access article distributed under the terms of the Creative Commons Attribution License (http://creativecommons.org/licenses/by/4.0/), which permits unrestricted use, distribution, and reproduction in any medium, provided the original work is properly cited.

**Session Chair**: Loren Martin, PhD, Assistant Professor, Department of Psychology, University of Toronto, Toronto, ON, Canada, lj.martin@utoronto.ca, @_ljmartin

**Symposium Abstract**: Cognitive factors are known to facilitate or inhibit pain perception. Salient painful stimuli involuntarily direct our attention to the source of the pain, resulting in increased pain, while pain memories can transition pain from an acute to a chronic state. Contrary to this, engaging in a cognitively demanding task reduces pain. The mechanisms underlying this trade-off interaction and its effect on pain modulation are less clear. This symposium will be focused on a multimethod approach to understand the bidirectional relationship between pain and cognition following three research lines:

1. We will examine the interaction between pain, cognition and motivation regarding the role of reward in performing a cognitive task, cognitive effort and resource allocation on pain perception. The value of a reward modulates the amount of cognitive effort and resources allocated to specific tasks, which in turn modulates pain perception.
2. Discuss the effect of neuromodulation on cognition and pain inhibition in healthy adults by exploring ways through which cognition can be improved and how this improvement influences pain perception.
3. Introduce novel paradigms of context-dependent pain modulation that are being implemented to study pain memory at the neurobiological level. These models may provide a better understanding of pain hypersensitivity and pain relief.

These presentations will provide a better understanding of the neural and psychological mechanisms underlying the interaction between pain and cognition. A better understanding of this bidirectional interaction can help in the development of improved interventions for individuals with pain.

### Speaker 1 Abstract Title: The role of value and cognitive resource availably in the trade-off between pain and cognitive effort

**Speaker 1**: Mathieu Roy, PhD, Assistant Professor, Department of Psychology, McGill University, Montreal, QC, Canada, mathieu.roy3@mcgill.ca

**Speaker 1 Abstract**: Pain acts as an alarm system; it rapidly disrupts ongoing activities to draw our attention towards sources of potential injury. However, pain may not always be the top priority, in which case it must be inhibited to allow us to focus on more important tasks. In order to examine this trade-off between pain and task performance, we conducted a series of experiments in which we asked our participants to perform a difficult working memory task (n-back task) while receiving painful thermal stimuli. In a first experiment (n = 41) we showed that inter-individual differences in threat sensitivity and executive functioning predicted the trial-by-trial trade-off between pain and task performance. In a second experiment (n = 40), we demonstrated that rewarding participants for their performance amplified the distracting effects of the task on pain, suggesting that cognitive effort is likely to be the determining factor in task analgesia. Finally, in one last study (n = 60), we depleted cognitive resources for our pain & working memory task by asking our participants to first perform a long and difficult cognitive test (O-span task; 40 min). Resource depletion abolished the effects of distraction on pain, supporting the idea that pain and cognitive functions share limited resources. Altogether, our results indicate that pain competes with other valued goals for access to executive resources, and that the trade-off is gated by the availability of cognitive resources and the value of pain relative to other goals.

### Speaker 2 Abstract Title: Improving working memory and pain inhibition in young and older persons using neuromodulation of left dorsolateral prefrontal cortex

**Speaker 2**: Zoha Deldar, PhD candidate, Université de Montréal/Université du Québec à Trois-Rivières, Anatomy, Trois-Rivières, QC, Canada, Zoha.deldar@uqtr.ca, @Zoha_Deldar

**Speaker 2 Abstract**: Effective attentional control of pain not only depends on the disengagement of attention from pain but also on the allocation of cognitive resources to maintain attention on the processing of task-relevant information unrelated to pain. Directing attention away from painful stimuli is under the control of working memory (WM) that allows the selection of task-relevant information and directing attention towards task execution. However, top-down inhibition of nociceptive activity and pain may be altered in patients with chronic pain and normal aging due to decreased WM. No therapeutic intervention has been proposed to alleviate this reduction in WM performance. Transcranial Direct Current Stimulation (tDCS) is a promising method in this regard since anodal tDCS of the left dorsolateral prefrontal cortex (DLPFC) was shown to improve WM performance. This talk will focus on the improvement of WM and pain inhibition by WM using tDCS in healthy young and older persons. Results of two experiments on forty healthy young volunteers and fifteen healthy older persons indicate that neuromodulation of left DLPFC enhances pain inhibition by improving WM in both groups. However, the nociceptive flexion reflex was not modulated by WM enhancement indicating that improvement of pain inhibition by WM using tDCS is independent of descending modulation pathways. These studies further our understanding of the interaction between cognition and pain, which has important implications for alleviating pain in patients affected by chronic pain.

### Speaker 3 Abstract Title: Learning models of pain and pain relief

**Speaker 3**: Loren Martin, PhD, Assistant Professor, Department of Psychology, University of Toronto, Toronto, ON, Canada, lj.martin@utoronto.ca, @_ljmartin

**Speaker 3 Abstract**: In humans, the cognitive processes of how an individual processes expectations and integrates different psychological elements plays an important role in shaping pain perception. For instance, in the clinic, when pain is anticipated, patients often report heightened pain sensations. Thus, behaviours associated with pain may not be intrinsic to the stimulus of pain, but may be a response to cognitive processing and external cues. Throughout this talk, I will describe novel animal models that we have been using to study the influence of conditioning on the “memory for pain” and the ‘relief of pain. We have also made considerable efforts within this domain to translate these findings to people. Further, our recent data show that targeted inhibition of “*memory-related”* proteins abolishes contextual pain memory in mice and through the use of pharmacological learning, we have shown that mice learn to associate environmental cues with pain-relief. The expectation of pain-relief activates specific neural patterns that are strikingly similar to the placebo response. These models provide a new means for studying the relationship between pain and memory by examining the influence of cognitive and pharmacological reinforcers, which will greatly enhance our understanding of the top-down modulation of pain processing.

**Learning Objective 1**: To examine the impact of reward on pain perception and cognitive task performance

**Learning Objective 2**: To examine the effect of neuromodulation in enhancing cognitive performance and pain inhibition in healthy young and old persons

**Learning Objective 3**: To explore the neurobiological mechanisms of pain memory, learning and conditioning through the use of novel animal and human paradigms

## Genes, Environments and Development in Pain: Crossing the Translational Divide

Marco Battaglia, Yves De Koninck, Steven Miller, and Simon Beggs

**CONTACT** Marco Battaglia marco.battaglia@camh.ca

© 2019 The Author(s). Published with license by Taylor & Francis Group, LLC.

This is an Open Access article distributed under the terms of the Creative Commons Attribution License (http://creativecommons.org/licenses/by/4.0/), which permits unrestricted use, distribution, and reproduction in any medium, provided the original work is properly cited.

**Symposium Abstract:** This symposium will address the roles of genetic and environmental factors that influence risk for pain, and the possible gene-environment interplay. Special emphasis will be put on the developmental years: how early-life adversities and exposure to moderately harmful stimuli can modify the perception of pain in a stable manner, and influence the risk for prospective pain syndromes.

This symposium brings together researchers and clinicians from both the human and the experimental fields, and will showcase investigations “*from preclinical to human, and back*”.

At the end of the symposium the listener will be able to appreciate how genetic and environmental factors influence pain early in life, how these processes likely unfold in a dynamic interplay, and how some preclinical data can be transferred to early risk identification and treatment applications in man.

### Speaker 1 Abstract Title: Genes and Environment in Adolescent Pain: Concepts and Research Strategies

**Speaker 1**: Marco Battaglia, MD, Centre for Addiction & Mental Health Division of Child Youth and Emerging Adult Programme & Department of Psychiatry University of Toronto ON, Canada, marco.battaglia@camh.ca

**Speaker 1 Abstract**: This presentation will introduce some fundamentals of genetic epidemiology, applied to young people and their proclivity to experience pain. How general population twin and family studies can reveal the degree of genetic and environmental influences on a variety of phenotypes including pain, and resolve the nature of comorbidity, such as the covariation between pain and anxiety/depression. Why gene-environment interplay matter, and how they can be studied in man and animals.

### Speaker 2 Abstract Title: Early interference with parental cares and altered nociception: learning from preclinical modelling

**Speaker 2**: Yves De Koninck, PhD CERVO Brain Research Centre & Laval University Quebec City Quebec, Canada yves.dekoninck@neuro.ulaval.ca

**Speaker 2 Abstract** This presentation will illustrate how early-life adversities can be modelled in preclinical paradigms to study altered nociception and pain. How interference with early maternal cares can induce multiple stable phenotypic changes including altered nociception and anxiety. How these responses result in part from gene-environment interplay that can be documented as epigenetic changes in animals and man. How these data can inform search for new treatments.

### Speaker 3 Abstract Title: The Early Environment of Preterm Newborns: Implications of Pain for Brain Development

**Speaker 3**: Steven Miller, MD The Hospital for Sick Children and The University of Toront, Department of Paediatrics, Toronto, Ontarion, Canada, steven.miller@sickkids.ca

**Speaker 3 Abstract**: Very preterm human neonates are exposed to numerous invasive procedures as part of life-saving care. Evidence suggests that repetitive neonatal procedural pain precedes long-term alterations in brain development. In this presentation we will examine how early exposure to painful stimuli during a period of rapid brain development, before pain modulatory systems reach maturity, predicts pronounced changes in thalamic development, and thereby cognitive and motor function. Early exposure to repetitive procedural pain in very preterm neonates may disrupt the development of regions involved in somatosensory processing, leading to poor functional outcomes. Furthermore, recent evidence suggests that certain analgesic and sedative medications that are commonly used in the neonatal period predict regionally specific alterations in brain development. More optimal pain management in the preterm neonate is an opportunity to enhance brain health and neurodevelopmental outcomes.

### Speaker 4 Title: Early-life pain experiences and their implications for persistent pain in adult life

**Speaker 4:** Simon Beggs, PhD UCL Great Ormond Street Institute of Child Health, London, UK. S.beggs@ucl.ac.uk

**Speaker 4 Abstract**: Considerable development of sensory systems occurs postnatally, with modality-specific interaction with the environment necessary for fine-tuning of sensory circuits in the central nervous system. Disruption of these developing circuits, e.g., through surgical injury, can instil long-lasting changes in sensory processing such that responses to painful stimuli in later life are exaggerated. Key to these effects are the interactions between the developing nervous and immune systems. Neuroimmune signalling is known to be a crucial mediator of nerve-injury induced chronic pain in adult animals. Here the postnatal development of spinal microglia and their interactions with local somatosensory circuitry will be described. Surgical injury in early postnatal life in a rodent model, using the hindpaw incision model, reveals a role for microglia in the subsequent priming of adult pain responses. Furthermore, MRI-based morphometry reveals the influence of early life pain exposure on structural changes in the brain following adult surgical incision. Intriguingly, inhibition of microglia reveals mechanistic differences in the priming effect between male and females. Exposure to painful events in a critical period of early postnatal life may represent a key risk factor in the development of persistent post-surgical pain in adulthood, with key differences in mechanism present between sexes.

**Learning Objective 1**: Learn about how to study genetic and environmental influences on pain early in life in human populations and pre-clinically;

**Learning Objective 2**: How these processes likely unfold in a dynamic interplay, and how some preclinical data can be transferred to early risk identification and treatment applications in man

**Learning Objective 3**: How early environment may affect brain development, pain proclivity, and pain persistence in adult life.

## Cannabis in Clinical Practice: Current and Future State

Lori Montgomery, G. Michael Allan, Barry D Kurtzer, and Hance Clarke

**CONTACT** Lori Montgomery Lori.montgomery@ahs.ca

© 2019 The Author(s). Published with license by Taylor & Francis Group, LLC.

This is an Open Access article distributed under the terms of the Creative Commons Attribution License (http://creativecommons.org/licenses/by/4.0/), which permits unrestricted use, distribution, and reproduction in any medium, provided the original work is properly cited.

**Symposium Chair**: Lori Montgomery, MD CCFP FCFP, Clinical Associate Professor, Cumming School of Medicine, Departments of Family Medicine and Anesthesiology, Perioperative and Pain Medicine, Calgary, Alberta, Lori.montgomery@ahs.ca, @_LoriMontgomery

**Symposium Abstract**: As we begin to determine how medical use of cannabis fits into the context of legalization, we have an opportunity to re-examine the existing evidence and refine our conversations with patients. This workshop will address three key issues: what does the evidence currently tell us about the role of cannabis in chronic pain, and how do we best share this with patients? What advice can we give our patients who are currently using cannabis (whether authorized or not) regarding impairment? What does the basic science tell us about the potential of cannabinoid medications, and where might research lead in the future? The presentations will be followed by a panel Q&A with all speakers.

### Speaker 1 Abstract Title: Is it high time for medical cannabis: critical thinking about the evidence in chronic pain

**Speaker 1**: G. Michael Allan, BSc, MD, CCFP, Director, Programs and Practice Support, College of Family Physicians of Canada,Toronto, Canada, MAllan@cfpc.ca

**Speaker 1 Abstract**: Curiosity about cannabis is at an all-time high, and chronic pain patients ask about it almost daily. It is important that we respond in an evidence-informed way. This workshop will outline the existing evidence for cannabis in pain, and help to distinguish high from low quality evidence. It will provide tips and tools for translating the evidence into a patient-centred conversation about the role of cannabis in pain management.

### Speaker 2 Abstract Title: Cannabis and impairment

**Speaker 2**: Barry D Kurtzer, BSc, MD MRO (AAMRO), Senior Staff Advisor, MRO and Medical Programs (retired), Driver Check Inc., Ayr, Ontario, Canada, bkurtzer@drivercheck.ca

**Speaker 2 Abstract**: Many of our pain medications have the potential to cause impairment, and cannabis is no exception. This session will provide 1) guidance for workers, employers, and health care professionals on occupational implications for a patient who is considering using cannabis, 2) information on driving and other safety sensitive activities, and 3) a guide to assessing impairment in a patient who is currently using either recreational or medical cannabis.

### Speaker 3 Abstract Title: The promise of cannabinoids and future directions

**Speaker 3**: Hance Clarke MD PhD FRCPC, Staff Anesthesiologist, Director of The Transitional Pain Program Medical Director Pain Research Unit Department of Anesthesia and Pain Management, Toronto General Hospital Assistant Professor, University of Toronto, Toronto, Ontario, hance.clarke@utoronto.ca, @Drhaclarke

**Speaker 3 Abstract**: Developing knowledge about the basic science of cannabinoids gives us hope that this class of analgesics may provide benefit for people with pain. This presentation will describe the features of cannabinoids that suggest that they will play an important role with respect to therapeutic areas. A research and development pathway will be the cornerstone for novel products and this presentation will outline ongoing basic and clinical research with the aims of demystifying the mechanisms of action, reliability and standardization of products in the years ahead.

**Learning Objective 1**: develop a patient-centred approach to discussing the evidence for cannabis in chronic pain

**Learning Objective 2**: frame a conversation with a patient about possible impairment as a result of cannabis use

**Learning Objective 3**: with reference to the basic science of cannabinoids, explain the potential for cannabis in pain management, and consider possible avenues for future research

## Let’s Talk about (Painful) Sex!

Paul Yong, Natasha Orr, Kate Wahl, and Lana Barry

**CONTACT** Paul Yong paul.yong@vch.ca

© 2019 The Author(s). Published with license by Taylor & Francis Group, LLC.

This is an Open Access article distributed under the terms of the Creative Commons Attribution License (http://creativecommons.org/licenses/by/4.0/), which permits unrestricted use, distribution, and reproduction in any medium, provided the original work is properly cited.

**Symposium Chair**: Dr. Paul Yong; MD, PhD; B.C. Women’s Hospital and Health Centre & Vancouver General Hospital, Obstetrics and Gynecology, Vancouver, BC, Canada, paul.yong@vch.ca, @PelvicPainEndo

**Symposium Abstract**: As many as 60% of women report experiencing sexual pain in their lifetime. This symptom negatively impacts psychosocial wellbeing, intimate relationships, and quality of life. Despite these sequalae, female sexual pain is under-researched and is often dismissed or mismanaged. The objective of this symposium is to summarize the pathophysiology and treatment of sexual pain in the context of clinical practice, research, and the patient experience. First, patient advocate Lana Barry will share her experience with sexual pain and highlight the importance of patient partners in research. Next, Dr. Paul Yong will discuss the etiology, diagnosis, and management of sexual pain. Finally, Natasha Orr and Kate Wahl will present quantitative and qualitative approaches to the investigation of female sexual pain.

### Speaker 1 Abstract Title: The Journey from Pain to Advocacy: A Patient Partner Experience

**Speaker 1**: Lana Barry, MEd, University of Victoria, Centre on Aging, Vancouver, BC, Canada, lbarry@uvic.ca, @selfcare4u

**Speaker 1 Abstract**: In 2012, Lana Barry was in a motor vehicle accident that resulted in genital pain that worsened in the months following the collision. Lana will outline the impact this pain had on her well-being, the toll of being told her pain was “all in her head,” and the process that lead to her diagnosis with provoked vestibulodynia. Next, Lana will describe how participating in a study of cognitive therapy and mindfulness for treatment of her condition lead her to develop a passion for self-advocacy. Finally, Lana will discuss her collaboration in the #ItsNotInYourHead knowledge dissemination campaign as well as her current work to promote the idea of female sexuality and health as a vital part of identity. Following her presentation, the audience will break into small groups and discuss how patients can collaborate on their research and what barriers and facilitators to this participation exist in their work.

### Speaker 2 Abstract Title: One Size Does NOT Fit All: A Multi-disciplinary Perspective on the Pathophysiology and Treatment of Female Sexual Pain

**Speaker 2**: Dr. Paul Yong; MD, PhD; B.C. Women’s Hospital and Health Centre & Vancouver General Hospital, Obstetrics and Gynecology, Vancouver, BC, Canada, paul.yong@vch.ca, @PelvicPainEndo

**Speaker 2 Abstract**: Dr. Paul Yong is a staff gynecologist and researcher specializing in sexual pain in endometriosis. He will describe two types of sexual pain – superficial dyspareunia and deep dyspareunia – and the contribution of gynecological conditions, comorbidities, and central sensitization to these types of pain. Dr. Yong will also provide insights into multidisciplinary care for these two types of sexual pain, and present relevant case studies and prospective outcome studies.

### Speaker 3 Abstract Title: Two Half of a Whole: Quantitative and Qualitative Methods in Female Sexual Pain Research

**Speaker 3**: Kate Wahl, BSc, University of British Columbia, School of Population and Public Health, Vancouver, Canada, kate.wahl@cw.bc.ca, @katejwahl; Natasha Orr MSc, University of British Columbia, Department of Obstetrics and Gynecology, Vancouver, Canada, natasha.orr@cw.bc.ca, @NatashaLeighOrr

**Speaker 3 Abstract**: The International Association for the Study of Pain emphasizes that pain is subjective, moreover pain is experienced differently by women and men. This makes female sexual pain a difficult phenomenon to measure. To exemplify this challenge, Natasha Orr and Kate Wahl will present qualitative and quantitative approaches to investigating female sexual pain. In particular, Kate will describe current challenges with self-reported female sexual pain in the endometriosis population, principally discrepancies between patient and provider understanding of sexual pain and the exclusion of LGBTQ2 individuals from current measures of sexual pain. Kate will share her experience interviewing women about their sexual pain and how this work will inform more appropriate measurement of sexual pain. Next, Natasha will describe her work to phenotype women with sexual pain based on the primary cause of their pain (i.e., endometriosis specific factors vs. central nervous system sensitization), using methods such as quantitative sensory testing and use of the central sensitization inventory. The session will end with an interactive activity focused on the integration of qualitative and quantitative methods.

**Learning Objective 1:** Realize the significant impact of female sexual pain from a patient perspective

**Learning Objective 2:** Understand the relationship between the etiology and multidisciplinary treatment of female sexual pain

**Learning Objective 3:** Learn about the examples of qualitative and quantitative methods in sexual pain research

## Should it be the sociopsychobio model of pain? Novel theoretical, clinical, and systems-level insights into social contexts of pain

Kenneth Craig 0000-0001-8063-2662, Whitney Scott 0000-0002-2529-9083, and Maria Hudspith

**CONTACT** Whitney Scott whitney.scott@kcl.ac.uk

© 2019 The Author(s). Published with license by Taylor & Francis Group, LLC.

This is an Open Access article distributed under the terms of the Creative Commons Attribution License (http://creativecommons.org/licenses/by/4.0/), which permits unrestricted use, distribution, and reproduction in any medium, provided the original work is properly cited.

**Symposium Chair**: Whitney Scott, PhD, King’s College London, Institute of Psychiatry, Psychology, and Neuroscience, London, UK, whitney.scott@kcl.ac.uk, @WhitneyJScott

**Symposium Abstract:** A proposal to update the definition of pain by Williams & Craig (2016) emphasizes the crucial role of social processes in the pain experience. However, within the biopsychosocial model of pain relatively less research has focused on social factors, as compared to biomedical or psychological factors. This session will argue for the need to place greater emphasis on the social context of pain from theoretical, clinical, and systems-level perspectives. Theoretical models of the role of social contexts and interpersonal processes in the experience of pain and related disability will be outlined. Clinical data will be discussed to demonstrate the impact of social stigma on chronic pain outcomes and current challenges and opportunities for managing stigma will be identified. The impact of systemic inequities and structural violence on pain experience will be highlighted. Opportunities to create more equitable health services through bringing together people from marginalized communities, healthcare providers, and researchers will be discussed. The session has the potential to advance theory and treatment development from both individual- and systems-level perspectives.

### Speaker 1 Abstract Title: Is pain a social experience?

**Speaker 1**: Kenneth Craig, PhD, University of British Columbia, Psychology, Vancouver, BC, Canada, kcraig@psych.ubc.ca

**Speaker 1 Abstract**: Abundant data confirms social contexts as powerful determinants of pain and related disability. The social environment controls: risks of exposure to pain, e.g., accidents, workplace risk and interpersonal violence; how people communicate distress to others, e.g., use of speech and efforts to convince others of the legitimacy of distress; the consequences of pain for participation in work, school, family and friendship activities; and how care is delivered, e.g., inadequate care and inappropriate response. Agreement on the importance of these determinants is considerable; more contentious would be the argument that pain is a social experience. Proponents of pain as exclusively a sensation fail to recognize psychosocial processes. It has been proposed that defining features of pain extend beyond sensory and emotional factors to include cognitive and social components, with the IASP President recently striking a task force to consider updating its definition of pain. Evolution dictated roles for sociality in nonhuman animals and humans decidedly have social brains; human sensitivities to painful experiences of others and the role of executive processing in negotiating optimal care when in pain implicate social thinking when in pain. Social learning attaches emotional significance and meaning to pain as children become socialized in familial and ethnic contexts. Recognizing that “the dysphoria of social rejection is integral to the experience of pain (Carr, 2018)” is fundamental to understanding increasingly important social systems caring for chronic pain patients distinguishable from traditional medical care. Attention to social determinants, including in pain education, contributes to innovative care for patients.

### Speaker 2 Abstract Title: The impact and management of stigma in people with chronic pain

**Speaker 2**: Whitney Scott, PhD, King’s College London, Institute of Psychiatry, Psychology, and Neuroscience, London, UK, whitney.scott@kcl.ac.uk, @WhitneyJScott

**Speaker 2 Abstract:** The potentially adverse impact of a social environment characterized by stigmatizing responses has been identified as an area of importance for research and clinical practice in chronic pain (De Ruddere & Craig, 2016). However, few studies have investigated the association between stigma and chronic pain outcomes. Moreover, research has not examined the extent to which experiences of stigma might change following interdisciplinary treatment for chronic pain. Research is also needed to understand the role of stigma in pain that occurs in the context of other highly stigmatized conditions, such as HIV. This presentation will discuss the psychometric properties of a previously developed questionnaire of enacted and internalized stigma for chronic illness in a sample of people with chronic pain. Associations between stigma and pain outcomes will be described. Data examining the magnitude of change in stigma following interdisciplinary treatment based on principles of Acceptance and Commitment Therapy for chronic pain will also be presented. Lastly, qualitative data describing the role of stigma in relation to neuropathic pain in people living with HIV will be discussed. Taken together, these data will be used to highlight the need for a multifaceted approach to address the stigma of chronic pain, which includes a focus on interpersonal and systems-level factors that perpetuate stigma.

### Speaker 3 Abstract Title: Understanding the role of systemic violence and structural inequities in the pain experience

**Speaker 3**: Maria Hudspith, MA, University of British Columbia, Pain BC, British Columbia, maria@painbc.ca

**Speaker 3 Abstract:** People living in marginalized conditions experience greater prevalence of painful medical conditions relative to non-marginalized people but access and utilize pain services less frequently. This project brought together a team consisting of researchers specializing in pain, trauma and violence-informed care and systemic discrimination to explore the intersection of these concepts. The team was supported through a Convening and Collaborating grant from the Michael Smith Foundation for Health Research and Pain BC, a collaborative non-profit organization working to improve the lives of people in pain in BC. The project engaged people from Indigenous, LGBTQ2S and newcomer/refugee communities to centre their lived experience with pain and marginalization. Members of the three communities participated in a series of focus groups as well as a knowledge translation gathering with clinicians, practice leaders and policy makers committed to furthering culturally safe and appropriate care. The project highlights the significant impact of systemic inequities and structural violence – including racism, homophobia and transphobia, and poverty - in the lives of people living with pain. It offers insight into the role of systemic factors in the pain experience and the importance of contextually-tailored care. Beyond the completion of the project, Pain BC will advance integration of the findings into practice, program development and policy through clinical education, health systems redesign initiatives and provincial policy.

**Learning Objective 1**: Upon attending this symposium, attendees will recognize the importance of social features of pain experience for understanding pain, pain education and innovative interventions.

**Learning Objective 2**: Upon attending this symposium, attendees will have an understanding of how stigma relates to chronic pain outcomes and approaches to managing stigma.

**Learning Objective 3**: Upon attending this symposium, attendees will have knowledge of the significant impact of systemic inequities and structural violence in the lives of people living with pain.

## Trauma-Related Symptoms Associated with Chronic Pain, Traumatic Injury, and Major Surgery in Youth and Adults: Neurobiological, Psychological and Public Health Perspectives

Hance Clarke 0000-0003-4975-3823, Jillian Vinall, Joel Katz 0000-0002-8686-447X, and Melita Giummarra

**CONTACT** Hance Clarke Hance.Clarke@uhn.ca

© 2019 The Author(s). Published with license by Taylor & Francis Group, LLC.

This is an Open Access article distributed under the terms of the Creative Commons Attribution License (http://creativecommons.org/licenses/by/4.0/), which permits unrestricted use, distribution, and reproduction in any medium, provided the original work is properly cited.

**Symposium Chair**: Hance Clarke, MD, FRCPC, PhD, Toronto General Hospital, Department of Anesthesia and Pain Management, Toronto, ON, Canada, Hance.Clarke@uhn.ca, @Drhaclarke

**Symposium Abstract**: Globally, pain, mental health conditions and trauma lead to some of the greatest burden of disability across the lifespan. Understanding the mechanisms and manifestations of these problems is therefore a major public health priority to enable us to develop and deliver more effective and timely interventions to the right person at the right time. In this symposium, Dr Jillian Vinall will first discuss the co-occurrence of post-traumatic stress disorder symptoms in youths with chronic pain, and will present novel insights into neurobiological mechanisms associated with varying levels of PTSD symptoms in youths with chronic pain. Second, Dr. Joel Katz will discuss the role of sensitivity to pain traumatization and anxiety-related disorders in the manifestation of persistent pain both before and after major surgery. Sensitivity to pain traumatization describes the propensity to develop anxiety-related responses to pain that are similar to traumatic stress reactions, but are specific to pain as the traumatic experience. Finally, Dr Melita Giummarra will provide an overview of the prevalence and trajectories of pain and mental health problems after traumatic injury in adolescents through to older adults using population-level trauma registry data from Victoria, Australia. These neurobiological, psychological and population level modelling insights have significant implications for the delivery of early, timely, appropriate and effective interventions across the lifespan. We will therefore highlight important policy implications for improved delivery of services and treatments for pain and mental health that might ultimately lead to reductions in the global burden of pain and mental health conditions.

### Speaker 1 Abstract Title: PTSD symptoms and chronic pain in youth: shared neurobiology as a mutually maintaining mechanism

**Speaker 1**: Dr. Jillian Vinall, PhD, University of Calgary, Anesthesia, Calgary, Alberta, Canada, jillian.vinall@ucalgary.ca, @Jillian_Vinall

**Speaker 1 Abstract**: Chronic pain (pain for ≥3 months) is alarmingly prevalent in adolescence (15–40% of youth), poses enormous costs to society, and can lead to persistent pain problems and mental health conditions into adulthood. Youth with chronic pain report having a greater number of traumatic events early in life compared to those without chronic pain, and this is associated with higher posttraumatic stress disorder (PTSD) symptoms. PTSD is a mental health condition characterized by prolonged distress following exposure to a traumatic event (e.g. injury, sexual violence). PTSD symptoms and chronic pain have been found to co-occur at high rates in both adolescent and adult samples (10–80%), and are linked to heightened impairment and disability. Comorbid chronic pain and PTSD has been explained by the presence of shared neurobiology. There are several regions within the central nervous system where nociceptive signals and responses to threat converge and interact to potentiate neural activation. However, the underlying neurobiology of comorbid pediatric chronic pain and PTSD has not been empirically examined. To examine the relationship between PTSD and chronic pain, we will present new findings from 30 youth (age 10–18 years) with chronic pain, including state-of-the-art neuroimaging data (i.e. task-based functional magnetic resonance imaging) and will discuss neural activation patterns of those individuals with varying levels of PTSD symptoms. Given the heightened impairment experienced by youth with comorbid chronic pain and PTSD symptoms, research is critically needed to examine neuronal mechanisms, and inform the development of targeted interventions to improve pain trajectories and health outcomes.

### Speaker 2 Abstract Title: Sensitivity to Pain Traumatization: Links between Trauma and Pain in Surgical Patients and Patients with Anxiety Disorders

**Speaker 2**: Joel Katz, PhD, York University, Psychology Department and Toronto General Hospital, Department of Anesthesia and Pain Management, Toronto, ON, Canada, jkatz@yorku.ca, @joeldkatz

**Speaker 2 Abstract**: Sensitivity to pain traumatization describes the propensity to develop anxiety-related somatic, cognitive, emotional, and behavioral responses to pain that resemble features of a traumatic stress reaction. The Sensitivity to Pain Traumatization Scale (SPTS) has been developed to measure this construct. Data from patients undergoing major surgery show that preoperative SPT scores, but not posttraumatic stress symptom scores, are significantly higher in patients with persistent pain versus those without persistent pain both before, and one-year after, surgery. These findings argue for SPT as a distinct construct separate from post-traumatic stress disorder. Most of what we know about the relationship between pain and trauma comes from samples of chronic pain patients who have been queried on trauma experiences. Less is known about pain in people with diagnosed anxiety disorders. This presentation will present SPTS scores and other psychosocial constructs from patients in the Transitional Pain Service at the Toronto General Hospital before and 6 months after undergoing major surgery. It will also present similar data from patients diagnosed with various anxiety disorders (generalized anxiety disorder, social phobia, pain disorder with and without agoraphobia). The presentation will focus on the common underlying factors that place people at risk of developing comorbid chronic pain and anxiety.

### Speaker 3 Abstract Title: Pain and mental health after injury: Who experiences persistent problems, and what role might early interventions have?

**Speaker 3**: Dr Melita Giummarra, BA (honours), PhD, School of Public Health and Preventive Medicine, Monash University, Melbourne, Victoria, Australia, Melita.giummarra@monash.edu, @melita.giummarra

**Speaker 3 Abstract**: Persistent pain is common after traumatic injury, and frequently co-occurs with mental health and emotional problems including anxiety, depression, post-traumatic stress disorder and suicidal ideation. This presentation will provide an overview of the predominant trajectories of pain and mental health problems after traumatic injury from population and registry-based studies from Victoria, Australia. These studies have shown that up to two thirds of people experience persistent or worsening problems with pain or mental health in the first two years after transport-related major trauma; however, few people receive treatments for those problems, especially those living in rural and regional areas. This presentation will therefore emphasize how we should use these population-level insights to improve the trauma and health systems to enable the proactive and timely delivery of treatments for pain and mental health across the care continuum to reduce the burden of injury.

**Learning Objective 1**: To better understand the neurobiological mechanisms underlying the development and maintenance of chronic pain and comorbid posttraumatic stress symptoms in youth.

**Learning Objective 2**: To better understand the psychosocial constructs underlying the risk of developing comorbid chronic pain and anxiety disorders.

**Learning Objective 3**: To provide an understanding of the predominant trajectories of pain and mental health over the first two years following injury, which can be used to proactively deliver timely and effective treatments to reduce the burden of injury.

## Pain in Cancer Survivorship: Applying a Lifespan Approach to Better Understand an Understudied Problem

Nicole M. Alberts, Fiona Schulte, Lynn R. Gauthier, and Myriam Asri

**CONTACT** Nicole M. Alberts nicole.alberts@stjude.org

© 2019 The Author(s). Published with license by Taylor & Francis Group, LLC.

This is an Open Access article distributed under the terms of the Creative Commons Attribution License (http://creativecommons.org/licenses/by/4.0/), which permits unrestricted use, distribution, and reproduction in any medium, provided the original work is properly cited.

**Symposium Chair**: Nicole M. Alberts, PhD, St. Jude Children’s Research Hospital, Department of Psychology, Memphis, Tennessee, USA, nicole.alberts@stjude.org, @NicoleMAlberts

**Symposium Abstract**: Advances in early detection and treatment have dramatically increased both pediatric and adult-onset cancer survival rates. Nonetheless, long-term treatment-related morbidity, also referred to as late effects, are common among survivors. Moreover, these effects can be disabling and life threatening. As the number of cancer survivors continues to grow and the population ages, it is likely that the burden of late effects on both the individual and society will also continue to increase. Despite the prevalence of pain during and after cancer treatments and its impact on functioning and quality of life, pain has remained understudied relative to other late effects. In recognition of this overlooked area, increasing calls have recently been made to bring attention to pain among survivors. Utilizing a lifespan and developmental approach, this symposium aims to amplify this call, and provide an overview of work examining pain in cancer survivorship among child, adolescent, young adult, and older adult populations. First, results of a qualitative study examining the pain narratives of pediatric survivors of childhood cancer will be described. Next, findings pertaining to the prevalence, predictors, and functional outcomes of pain among adolescent and young adult survivors of childhood cancer will be presented. Finally, results of a longitudinal study examining age-related patterns in acute and chronic pain pain among breast cancer survivors will be summarized. Clinical implications of these findings as well as future directions for advancing the field of pain and cancer survivorship will be discussed.

### Speaker 1 Abstract Title: The pain of survival: An examination of pain narratives in long-term survivors of childhood cancer and their caregivers

**Speaker 1**: Fiona Schulte, PhD, Department of Oncology, Division of Psychosocial Oncology Cumming School of Medicine, University of Calgary, Calgary AB, CANADA, fsmschul@ucalgaryca, @schultefiona

**Speaker 1 Abstract**: The population of long-term survivors of childhood cancer (LTSCC) is increasing rapidly, with 85% of adolescents surviving at least 5-years post-diagnosis. Yet, two thirds of these survivors will face significant long-term effects of their treatment, including pain. Compared to a sibling control group, LTSCC more than 15 years post-treatment, have been shown to report twice as much pain. Despite this, we do not understand much about the experience of pain in LTSCC. This study aims to qualitatively document survivor and parent accounts of the survivor’s experience of pain, the impact of that pain, and the meaning ascribed to pain, leading up to diagnosis and then into survivorship. Survivors of childhood-onset acute lymphoblastic leukemia (ALL), 8–21 years of age, and two years post-treatment, have been recruited in addition to one caregiver of each eligible participant (n = 20 dyads). Survivors and caregivers have independently completed qualitative interviews asking about their memory of pain, the impact of the pain, and the meaning of pain, anchored across three time points: prior to diagnosis, during active treatment, and following treatment completion. Interviews are currently being transcribed. Narratives are being coded in terms of affect (i.e., positive vs. negative) as well as content. Specific content of interest will include: fear of cancer recurrence, anxiety, pain catastrophizing, benefit-finding, optimism, moving forward/return to well-being, self-efficacy in pain control. Future research will explore the role of memory and trauma in the development of pain and the parent child experience. Clinical implications of this work will be discussed.

### Speaker 2 Abstract Title: Prevalence and functional consequences of pain in adolescent and young adult survivors of childhood cancer

**Speaker 2**: Nicole M. Alberts, PhD, St. Jude Children’s Research Hospital, Department of Psychology, Memphis, Tennessee, USA, nicole.alberts@stjude.org, @NicoleMAlberts

**Speaker 2 Abstract**: Childhood cancer survival rates have improved drastically over the past 50 years. Nevertheless, roughly two-thirds of childhood cancer survivors will develop at least one complication due to their cancer treatment, with one-third of survivors facing serious or life-threatening complications. Of these late effects, pain has been reported in up to 59% of adult survivors. In addition, it is likely that the presence of other late effects such as musculoskeletal conditions and fatigue further compound pain and pain-related disability among survivors. Despite these factors, little research has examined pain among adolescent and young adult (AYA) survivors of childhood cancer. This developmental period poses specific challenges to pain management – including the task of transitioning from pediatric to adult health care systems. However, it also presents a potentially fruitful period for intervention, such that pain is targeted prior to the transition to adulthood where it may become more entrenched. In this presentation, we will present data from over 500 AYA survivors of childhood cancer, stratified by developmental period (i.e., 12–17 years vs. 18–39 years), from a large survivorship clinic in the United States. The prevalence of pain as well as demographic, disease, and treatment factors associated with pain among AYA childhood cancer survivors will be described. To gain a better understanding of the impact of pain on AYA functioning, associations between pain and physical activity, social functioning, and cancer-related worry will also be discussed. Clinical implications and future research directions for the study of pain among AYA childhood cancer survivors will be presented.

### Speaker 3 Abstract Title: Age-related patterns in taxane-induced acute and chronic pain and other sensory symptoms among adult breast cancer survivors

**Speaker 3**: Lynn R. Gauthier, PhD, Université Laval, Department of Family and Emergency Medicine, Québec, QC, Canada, lynn.gauthier@crchudequebec.ulaval.ca, @docpeper

Myriam Asri, BScN, RN, Health Admin. MSc, PhD student, Université Laval, Department of Community Health, Québec, QC, Canada, myriam.asri.1@ulaval.ca

**Speaker 3 Abstract**: Breast cancer is the most common cancer among women. With the aging population and treatment advances, in the next decade, more than 2/3 of survivors will be older than 60. They may be forced to manage significant long-term treatment-related morbidity, in addition to other complex age-related health conditions. An important source of this morbidity may be chemotherapy-induced peripheral neuropathy (CIPN), characterised by painful and non-painful sensory symptoms in the periphery (e.g. numbness, tingling, or burning), and widespread musculoskeletal pain, both poorly understood effects of taxane-based chemotherapy for breast cancer. Although it has been suggested that older age is a risk factor, data are equivocal and most studies are not designed to investigate age. Moreover, chronological age may be a proxy for a host of age-related biopsychosocial risk factors yet to be investigated. This presentation will describe recent findings from a longitudinal study of age-related patterns in acute and chronic pain and sensory symptoms in adult women undergoing taxane-based treatment for breast cancer. Although age is not associated with acute and chronic pain intensity and nociceptive pain, compared to younger adults, older adults (≥60 years) experience greater chronic CIPN (β = .36, p = .05) and a greater increase in neuropathic pain immediately after treatment, which persists >3 months (p ≤ .04). Age-related patterns in risk factors, including clinical factors, peripheral nerve fibre functioning, and physical and psychosocial wellbeing, will be presented. Implications for tailored treatments and future research directions elucidating a lifespan-developmental model of pain and other sensory symptoms in adult cancer survivors will be discussed.

**Learning Objective 1**: To bring awareness to the problem of pain in cancer survivorship.

**Learning Objective 2**: To consider the influence of treatment/procedure, health, psychological, and developmental factors on pain among survivors.

**Learning Objective 3**: To describe the use of quantitative and qualitative research methods currently being applied to the study of pain in cancer survivorship.

## Interventional Procedures for the Management of Chronic Non-Cancer Pain

Ian Beauprie, Philip Peng, and Harsha Shanthanna

**CONTACT** Harsha Shanthanna shanthh@mcmaster.ca

© 2019 The Author(s). Published with license by Taylor & Francis Group, LLC.

This is an Open Access article distributed under the terms of the Creative Commons Attribution License (http://creativecommons.org/licenses/by/4.0/), which permits unrestricted use, distribution, and reproduction in any medium, provided the original work is properly cited.

**Symposium Chair**: Harsha Shanthanna MD, MSc, FRCPC, Associate Professor, McMaster University, Department of Anesthesia, Hamilton, ON, Canada; shanthh@mcmaster.ca

### Speaker 1 Abstract Title: Radiofrequency procedures for chronic pain: Mechanism, Evidence and Public health implications.

**Speaker 1**: Ian Beauprie, MD, FRCPC; Associate Professor, Dalhousie University, Department of Anesthesia, Pain Management and Perioperative Medicine, NS, Canada; i.beauprie@dal.ca

**Speaker 1 Abstract**: Radiofrequency procedures are commonly utilized for the management of chronic back and neck pain. Pain relief from RF procedures is based on microwave (heat) destruction of nerves. Evidence supporting the use of conventional RF is conflicting depending on recruited cohorts. In recent years there has been several variations in the delivery of RF energy to make it safer and also to improve the efficiency. Indications may expand to include large joints such as the knee. Back and neck pain affect a large proportion of the population, and these are perhaps the most lucrative procedures in pain medicine. We will describe modalities of RF treatment, the existing evidence, and the debate around patient selection. We will also discuss the important limitations and practice considerations in the use of RF procedures for chronic pain.

### Speaker 2 Abstract Title: Interventions for the management of Hip and Knee Joint Pain

**Speaker 2**: Philip Peng, MBBS FRCPC, Founder (Pain Med), Professor, University of Toronto, Department of Anesthesiology and Pain Management, Toronto, ON, Canada; Philip.Peng@uhn.ca

**Speaker 2 Abstract**: Hip and knee joint pain arthritis leading to chronic pain affects a large proportion of elderly patients. Although joint replacement is considered a definitive solution, many patients do not achieve satisfactory pain relief or not found to be appropriate candidates. Intraarticular injections have been performed with various agents with mixed results. Recently, there has been renewed focus on knee and hip joint innervation and consideration for radiofrequency interventions on these target nerves. Use of ultrasound allows precise identification of joint structures and nerves. In this talk we will summarize the available interventions and their limitations for hip and knee joint pain, describe recent findings from anatomical studies regarding their innervation, and propose considerations for future practice and research.

### Speaker 3 Abstract Title: Evidence Based Interventions for Chronic Pain: Present State and Future Directions

**Speaker 3**: Harsha Shanthanna, MD, MSc, FRCPC, Associate Professor, McMaster University, Department of Anesthesia, Hamilton, ON, Canada; shanthh@mcmaster.ca

**Speaker 3 Abstract**: As physicians, we all have a responsibility to practice medicine that is based on evidence. However, there are questions and criticism on the level and quality of evidence that exists to support most interventional pain treatments. Further, there are reports to suggest that there is disproportionate increase in the use of these techniques, in Canada and other developed countries. In this talk, we will critically appraise the existing evidence and the demonstrate the need to evaluate pain interventions by rigorously done clinical studies. We will also look at the challenges for conducting randomized control trials in interventional pain medicine, and potential considerations for adapting other study designs. Lastly, we will highlight the ongoing efforts to provide evidence informed guidelines for interventional pain practice in Canada.

**Learning Objective 1**: To understand the mechanisms of RF treatment; strategies to select patients; new indications and modalities; and the implications for health budgets as RF treatment becomes more widespread.

**Learning Objective 2**: To understand the limitations of existing treatments for hip and knee joint pain; appreciate the innervation of knee and hip joints and potential sensory targets for pain interventions; and to discuss the potential role of image guided nerve block and radio-frequency treatments for knee and hip joint pain.

**Learning Objective 3**: To understand the evidence behind commonly performed interventional pain treatments and their limitations; appreciate the need for clinical studies and guidelines to better inform clinicians to perform evidence-based interventions; and to be aware of the ongoing efforts to promote evidence based pain interventions in Canada.

## Social Mechanisms Underlying the Pain Experience: Novel Frameworks for Examining the Influence of Social Context

Loren Martin, Andrey Ryabinin, Kristen Jastrowski Mano

**CONTACT** Loren Martin lj.martin@utoronto.ca

© 2019 The Author(s). Published with license by Taylor & Francis Group, LLC.

This is an Open Access article distributed under the terms of the Creative Commons Attribution License (http://creativecommons.org/licenses/by/4.0/), which permits unrestricted use, distribution, and reproduction in any medium, provided the original work is properly cited.

**Session Chair**: Loren Martin, PhD, Assistant Professor, Department of Psychology, University of Toronto, Toronto, ON, Canada, lj.martin@utoronto.ca, @_ljmartin

**Symposium Abstract**: Pain is considered a personal experience, but it is, in fact, rarely private. Individuals’ behavioral responses to pain function to communicate distress to others in the environment, eliciting emotional reactions and caregiving actions that will in turn impact the sufferer’s pain experience. This symposium will highlight the importance of understanding the social context of pain from a mechanistic perspective and how social threat alters the pain experience and emotionality in general and chronic pain populations. Evidence from both the basic science and clinical perspectives will be presented, illustrating how pain experiences can impact social interactions and how reactions from others in the social environment and the environment itself impact the sufferer’s pain experience. Given the complex nature of social context and social interactions on pain sensitivity in humans and non-human animals, dissecting their integral role in mediating pain outcomes is critical. Our goal is to engage clinicians with pain neuroscientists to address how basic and clinical scientists can best address these complex questions. Thus, speakers will provide insight into (1) the fundamental mechanisms that engage the neural circuits responsible for pain modulation via social context, (2) the social transmission of pain sensitivity and lastly (3) how attentional biases to social threat may represent a critical mechanism underlying the co-occurrence of chronic pain and anxiety among chronic pain patients.

### Speaker 1 Abstract Title: Examining the neural circuits and molecular targets for the social modulation of pain

**Speaker 1**: Loren Martin, PhD, Assistant Professor, Department of Psychology, University of Toronto, Toronto, ON, Canada, lj.martin@utoronto.ca, @_ljmartin

**Speaker 1 Abstract**: It is well known that social context robustly affects pain levels and outcomes in chronic pain patients. Direct effects of varying social context on laboratory pain sensitivity have also been demonstrated but prove to be complex. It is of considerable surprise to many that social contexts and social interactions affect pain sensitivity in laboratory animals – but such observations have been made, and interest in the topic is growing. We have found that familiarity between mice is necessary for the social facilitation of pain behaviors and activation of “stress” receptors within “*affective*” brain regions prevents this enhancement. Additionally, we have also developed novel paradigms for social reunion behaviours that modulate and are modulated by pain. Here, we find that reunion between siblings causes analgesia to acute pain stimuli, whereas social interactions with a stranger result in hyperalgesia. However, mice experiencing inflammatory pain show no evidence of social analgesia and demonstrate reduced social grooming and approach behaviors following reunion. Neural activation and pharmacological interrogation of these behaviors will also be discussed. Overall, our models provide a new framework for studying the influence of social context on pain and give insight into the fundamental mechanisms that engage the neural circuits responsible for pain modulation via social context.

### Speaker 2 Abstract Title: Social transfer of hyperalgesia in rodents

**Speaker 2**: Andrey Ryabinin, Ph.D., Professor, Department of Behavioral Neuroscience, Oregon Health & Science University, Portland, OR, USA, ryabinin@ohsu.edu

**Speaker 2 Abstract**: Chronic pain not only affects patients but also influences the well-being of individuals surrounding them. Our experiments replicated a similar phenomenon in laboratory mice. Specifically, hyperalgesia in mice due to alcohol or morphine withdrawal or due to injection of an inflammatory agent resulted in hyperalgesia in experimentally-naïve mice that were housed in separate cages within the same room. Subsequent experiments demonstrated that such social transfer of hyperalgesia is not unique to mice, but also can be observed in prairie voles, rodents with a different social structure than mice. Perhaps surprisingly, the social transfer-induced hyperalgesia was not accompanied by increased activity of the hypothalamic-pituitary-adrenal axis or measures of anxiety. Similarly, this hyperalgesia was not attenuated by administration of a glucocorticoid inhibitor or an anxiolytic drug, suggesting that the transfer is specific to the sensation of pain and is not due to general arousal or stress. Investigations into the mode of communication underlying this social transfer pointed to the importance of olfactory cues. Using the olfactory preference test we demonstrated that cues associated with hyperalgesia are indeed aversive to mice, confirming the negative emotional valence of this experience. Experiments testing neural substrates of this hyperalgesia showed increased activation of anterior cingulate and anterior insula in the mice. Inhibition of anterior cingulate using chemogenetic approaches blocked social transfer- and alcohol withdrawal-induced hyperalgesia, indicating a crucial role for this brain region in this phenomenon. These findings not only demonstrate the need for critical evaluation of control conditions in preclinical studies, but also provide evidence for importance of social environment in the development of chronic pain.

### Speaker 3 Abstract Title: Attentional bias to social threat in pediatric chronic pain.

**Speaker 3**: Kristen Jastrowski Mano, Ph.D., Assistant Professor, Department of Psychology, University of Cincinnati, Cincinnati, OH, USA, manokn@ucmail.uc.edu

**Speaker 3 Abstract**: Children and adolescents with chronic pain experience high rates of co-morbid anxiety psychopathology, with upwards of 80% of chronic pain patients meeting criteria for an anxiety disorder based on structured diagnostic interviews. Attentional biases (ABs) – heightened attentional capture toward cues that are perceived as threatening and aversive – are involved in the etiology and maintenance of both anxiety disorders and chronic pain. Despite compelling evidence that youth with chronic pain experience elevated *social* anxiety and exhibit avoidance of threatening *social* contexts (including school and extracurricular activities), scant research exists examining ABs toward social threat in pediatric chronic pain. Thus, we investigated attentional biases to social threat among adolescents with and without chronic pain using utilizing novel eye tracking methods and reaction time tasks. Our data show clear differences between adolescent chronic pain patients’ responses to social threat as well as self-reported social anxiety symptoms compared to age- and gender-matched healthy controls. Drawing from a shared vulnerability model of chronic pain and anxiety, the link between attentional biases to social threat, fear-related avoidance and functional disability will be discussed. Research elucidating the specific processes that characterize attentional biases to social threat have important clinical implications for the development of intervention programs that aim to reduce anxiety among youth with chronic pain.

**Learning Objective 1**: To understand the neural circuits and molecular targets for the social modulation of pain.

**Learning Objective 2**: To understand the contribution of social environment to induction of pain and the role underlying neural circuits.

**Learning Objective 3**: Illustrate how attentional biases to social threat represent an important mechanism underlying the co-occurrence of chronic pain and anxiety.

## Ethical, Legal, and Social Dimensions of Chronic Pain: Considerations for Medical Assistance in Dying, the Overdose Crisis, and a National Pain Strategy

Daniel Z. Buchman 0000-0001-8944-6647, Jennifer A. Chandler, and Karen D. Davis 0000-0003-1879-0090

**CONTACT** Daniel Z. Buchman daniel.buchman@utoronto.ca

© 2019 The Author(s). Published with license by Taylor & Francis Group, LLC.

This is an Open Access article distributed under the terms of the Creative Commons Attribution License (http://creativecommons.org/licenses/by/4.0/), which permits unrestricted use, distribution, and reproduction in any medium, provided the original work is properly cited.

**Symposium Chair**: Daniel Z. Buchman PhD, MSW, RSW, Bioethicist and Clinician Investigator, University Health Network, Toronto, Ontario, Canada, daniel.buchman@utoronto.ca, @DanielZBuchman

**Symposium Abstract**: Chronic pain remains a major public health problem in Canada and globally. There are promising advances in science and technology that could improve the management of pain. North American society is also in the midst of an alarming rise in individual and population-level harms due to opioid-related overdoses. Efforts towards improving pain management as well as opioid-related morbidity and mortality have raised ethical, legal, and social questions for pain sufferers and their families, clinicians, scientists, and policymakers. Recent societal changes relevant to pain include a landmark Supreme Court of Canada decision, where the experience of pain and intolerable suffering featured prominently in the Court’s decision to permit eligible persons to request euthanasia. These social transformations exist alongside efforts to develop a National Pain Strategy for Canada. This Strategy will be instrumental in defining a Canadian approach for pain management, research, and education. In this symposium, we address the ethical, legal, and social dimensions of three timely issues that affect pain management, research, education, and policy in Canada. First, we discuss pain, suffering, and eligibility for euthanasia. Second, we examine the ethics of stigma, chronic pain, and substance use disorders in context of the overdose crisis. Finally, we explore how neuroethics should be considered and included in the creation of Canada’s first National Pain Strategy.

### Speaker 1 Abstract Title: Pain Syndromes, Suffering, and Canada’s New Medical Assistance in Dying Law

**Speaker 1**: Jennifer A Chandler, BSc, JD, LLM, Professor, Bertram Loeb Research Chair, Centre for Health Law, Ethics and Policy, Faculty of Law, University of Ottawa, Ottawa, Ontario, Canada, chandler@uottawa.ca, @jnfrchandler

**Speaker 1 Abstract**: The 2015 Carter decision of Canada’s Supreme Court radically changed Canadian law and society by removing criminal code impediments to medical assistance in dying under certain circumstances. One of the key eligibility requirements identified by the Court was the existence of enduring and intolerable suffering as a result of a grievous and irremediable medical condition. At least one Canadian with a conversion disorder was approved for euthanasia prior to the enactment of the federal legislation in 2016, which added further eligibility criteria whose practical effect was to make most people with psychiatric conditions ineligible for euthanasia in Canada. It is more likely that frail, elderly patients with pain conditions might be eligible. This talk will trace the treatment of pain syndromes in the evolving Canadian law, with particular attention to ethico-legal issues related to eligibility for euthanasia.

### Speaker 2 Abstract Title: Chronic Pain, Substance Use, and Stigma in Context of the Overdose Crisis

**Speaker 2**: Daniel Z. Buchman, PhD, MSW, RSW, Bioethicist and Clinician Investigator, University Health Network, Toronto, Ontario, Canada, daniel.buchman@utoronto.ca, @DanielZBuchman

**Speaker 2 Abstract**: Chronic pain and substance use disorders are highly stigmatized, and this stigma is intensified when both conditions occur concurrently. The contemporary opioid overdose crisis has exacerbated this dual stigma and has created a climate of distrust in chronic pain management. In this presentation, I explore how stigma manifests at the intersection of chronic pain and substance use on individual, social, and structural levels. I draw upon recent findings from qualitative interview studies and knowledge syntheses to describe the complex yet nuanced relationship between stigma, chronic pain, and substance use disorders. I also discuss how common clinical and policy approaches in response to the overdose crisis, such as opioid treatment agreements and prescription drug monitoring programs, may further intensify the stigma of pain sufferers.

### Speaker 3 Abstract Title: Neuroethics Considerations for a National Pain Strategy

**Speaker 3**: Karen D. Davis, PhD, FCAHS, Krembil Research Institute, Division of Brain, Imaging and Behaviour – System Neuroscience, Toronto, Ontario, Canada, karen.davis@uhnresearch.ca, @kren27

**Speaker 3 Abstract**: Efforts are underway to develop a National Pain Strategy that will shape a Canadian approach for pain management, research, and education. However, there has been little discussion about neuroethical and ethical societal issues that will arise from initiatives to shape healthcare, research and educational policy for the 21^st^ century. This talk will open up a discussion of these issues, including access to care and technologies, privacy, and protection of personal health information. As an illustrative example, I will discuss the issues associated brain decoding technologies and the collection of brain imaging data for the purposes of validating chronic pain (see Davis et al., Nature Rev Neurology 2017). Parallels will be drawn from experiences in issues arising from the development of genetic testing and policies that arose to protect personal genetic information.

**Learning Objective 1**: Understand the ethical and legal issues associated with pain syndromes and eligibility for medical assistance in dying;

**Learning Objective 2**: Recognize how chronic pain and substance use stigma may become intensified in context of the current overdose crisis;

**Learning Objective 3**: Explore how neuroethics issues should be considered in the development of a National Pain Strategy for Canada and the future of pain policy.

## Keeping the “I” in Pain: Theoretical, Methodological and Clinical Strategies for Integrating the Subjective Experience of Pain within Research and Practice

Timothy H. Wideman, Eloise Carr 0000-0003-1870-4244, and Stephen G. Henry

**CONTACT** Timothy H. Wideman timothy.wideman@mcgill.ca

© 2019 The Author(s). Published with license by Taylor & Francis Group, LLC.

This is an Open Access article distributed under the terms of the Creative Commons Attribution License (http://creativecommons.org/licenses/by/4.0/), which permits unrestricted use, distribution, and reproduction in any medium, provided the original work is properly cited.

**Symposium Chair**: Timothy H. Wideman, PT, PhD, McGill University, School of Physical and Occupational Therapy, Montreal, Quebec, Canada, timothy.wideman@mcgill.ca

**Symposium Abstract**: The “Holy Grail” for pain assessment research is often framed as an objective biomarker that can validate, or invalidate, the reported pain experience and guide clinical decision-making. The broader context for this quest, is a literature base that has historically emphasized the use of quantitative methodologies to study pain. Within this context, pain assessment strategies are typically focused on aspects of pain most readily communicated through numbers, such as pain intensity ratings or pain threshold levels. While quantitative pain measures are vital to understanding and targeting mechanisms and benchmarking management, they often overlook important attributes of the subjective experience, such as the personal context and meaning that shape our experiences of pain and suffering. This workshop aims to provide a novel perspective on the flipside of this historic trend by highlighting the inherent value of and need for qualitative methodologies that specifically address subjectivity related to pain. Presentations will provide theoretical, methodological and clinical perspectives on how to integrate personal language with standardized measures in order to better address the subjective experience of pain. Workshop presenters will speak from their diverse clinical backgrounds in physical therapy, nursing and medicine and research experience that draws on both qualitative and quantitative methodologies. Researchers and clinicians in the audience are expected to develop a new way of considering pain assessment that emphasizes the relative ability and value of different methodologies in addressing the subjective experience of pain.

### Speaker 1 Abstract Title: The Multi-modal Assessment of Pain (MAP) Model: A novel conceptual framework for further integrating the subjective pain experience within research and practice

**Speaker 1**: Timothy H. Wideman, PT, PhD, McGill University, School of Physical and Occupational Therapy, Montreal, Quebec, Canada, timothy.wideman@mcgill.ca

**Speaker 1 Abstract**: Pain is an enigmatic phenomenon that is challenging to treat and study. One challenging attribute is its subjective nature. Defined as a subjective experience, pain cannot be directly observed by those who aren’t experiencing it. Yet, clinicians and researchers rely upon observations and measures to assess and infer pain experienced by others. This raises the fundamental question of how the inherent subjectivity of pain can and should be addressed and integrated within its clinical assessment. This presentation will introduceThe Multi-modal Assessment of Pain (MAP) Model, which addresses this question by (1) specifying a root proxy for pain experience; (2) characterizing how different assessment methodologies relate to pain subjectivity; and (3) creating frameworks to further integratethe subjective pain experience within research and practice. The MAP model provides a rationale for why the qualitative words used to describe pain (i.e. the pain narrative) should be regarded as the best available root proxy for inferring pain and why comprehensive pain assessment should be regarded as both multi-dimensional and multi-modal (i.e. purposefully integrating both qualitative and quantitative assessment strategies). The MAP model offers a clinical framework that facilitates both a compassionate approach to assessment by validating pain reports, as well as a mechanism-based approach to management by integrating comprehensive assessment data. Its research framework shows how qualitative data can help characterize and contextualize quantitative pain measures. The presentation will conclude with a discussion of how The MAP model can help improve clinical training, inform ongoing debates on biomarkers and encourage new research on pain subjectivity.

### Speaker 2 Abstract Title: The added value of mixed methods research: Connecting and integrating the patient’s voice in pain research

**Speaker 2**: Eloise Carr, BSc(RN), MSc, PhD, University of Calgary, Faculty of Nursing, Calgary, Alberta, Canada, ecarr@ucalgary.ca

**Speaker 2 Abstract**: Mixed methods research (qualitative and quantitative methods) offers unique added value for pain researchers. Historically the integration of quantitative and qualitative methods in the same research study has been viewed with concern due their apparent incompatibility. However, there has been a growing interest in the field of mixed methods research that has found ways to bring these two world perspectives together to create a more meaningful understanding of the research enquiry. Despite the exponential growth in mixed methods research over the past 20 years, it remains relatively marginalized in pain research. This presentation aims to show how mixed methods research can be used to give the patient’s subjective pain experience a meaningful voice in research. The presentation is organized into three sections. The first section will provide a brief overview of the historical evolution of mixed methods research with its philosophical roots in the pragmatic paradigm. This paradigm brings the motivation for conducting research that is connected to changing or improving practice. The second section considers how to design a mixed methods study and the four foundational models frequently used. These are convergent, explanatory, exploratory and embedded. Each of these models will be illustrated with examples from the pain literature. The third section considers some of the challenges faced by mixed methods researchers, and how they might be addressed. Participants are expected to develop a new understanding of mixed methods research and its value for advancing research on the subjective experience of pain.

### Speaker 3 Abstract Title: Clinical strategies for evaluating the subjective nature of pain in primary care

**Speaker 3**: Stephen G. Henry, MD, MSc; University of California – Davis; Department of Internal Medicine, Sacramento, California, USA, sghenry@ucdavis.edu

**Speaker 3 Abstract**: This presentation discusses the clinical importance of eliciting patient narratives about pain and reviews empirically-based strategies for doing so. Evaluating patients’ experience with pain is important for understanding how pain affects patients’ function, emotional state, and social relationships during their everyday lives, particularly for chronic pain, and is often critical for formulating treatment plans in challenging clinical scenarios. Data and preliminary results from two ongoing studies about patients taking opioids for chronic pain – a common and frequently challenging scenario – will be reviewed to illustrate practical strategies for eliciting patient narratives about pain. One study examined the opioid tapering experience for patients with chronic neck and back pain. The second study interviewed patients and physician trainees to identify training needs for discussing chronic pain and opioids in primary care and identify effective communication strategies for doing so. Patients in both studies identified the desire for clinicians to ask questions that allowed patients to the opportunity to tell stories about their pain. Study data will be used to provide suggestions for how clinicians can elicit patients’ narratives and subjective experiences of pain by asking “narrative-type” questions, and also how they can use patients’ stories to elicit clinical information for formulating mutually acceptable pain treatment plans. Eliciting patients’ experiences with pain also shows patients that clinicians take patients’ pain seriously, gathers clinically important information, and builds rapport with patients. Teaching trainees to elicit patient narratives and evaluate patients’ subjective experience of pain should thus be an important component of clinical training.

**Learning Objective 1**: Develop a new conceptual framework for understanding how the inherent subjectivity of pain influences its assessment and management.

**Learning Objective 2**: Understand how to effectively integrate qualitative and quantitative research methodologies to better access novel aspects of the subjective experience of pain.

**Learning Objective 3**: Develop practical clinical skills for evaluating and addressing patients’ subjective experiences of pain within challenging primary care settings.

## Beyond Pediatric Pain: The Mutual Influence of Child Pain and Cognitive, Emotional and Social Development

Maria Pavlova, Ruth E. Grunau 0000-0002-5428-9212, and Rebecca Pillai Riddell 0000-0003-3990-3680

**CONTACT** Rebecca Pillai Riddell rpr@yorku.ca

© 2019 The Author(s). Published with license by Taylor & Francis Group, LLC.

This is an Open Access article distributed under the terms of the Creative Commons Attribution License (http://creativecommons.org/licenses/by/4.0/), which permits unrestricted use, distribution, and reproduction in any medium, provided the original work is properly cited.

**Symposium Chair**: Dr. Rebecca Pillai Riddell, PhD, York University, Department of Psychology, Hospital for Sick Children, University of Toronto, Toronto, Ontario, rpr@yorku.ca, @drbeccapr

**Symposium Abstract**: Pain in childhood is prevalent. Painful medical procedures (e.g., surgeries, immunizations), everyday cuts and bruises, and acute or chronic illness-related pain are a normative part of children’s lives from the first days. Nociception shapes children’s and caregivers’ behavioural and psychosocial reactions to pain. Painful experiences of infancy and early childhood produce a cascade of effects on children’s brain development and long-term developmental outcomes. At the same time, nociception and pain experiences are powerfully influenced by cognitive and psychological factors that undergo extensive changes in early childhood. For instance, children’s rapidly developing language, communication skills, and autobiographical memory significantly alter parent-child verbal exchanges about the immediate and past pain. Social context, a key component in the experience of pain, is particularly robust in early childhood with parents exerting considerable influence on immediate pain experiences and their aftermath. For example, certain parent behaviours may increase or, on the contrary, alleviate infant distress during painful medical procedures. Further, parents may reduce detrimental long-term effects of pain-related distress following hospitalization at the neonatal intensive care unit. The proposed symposium will examine how children’s cognitive, psychological, and social development and pain experiences mutually shape and influence each other within the context of changing parent-child verbal and non-verbal interactions. The panel includes an interdisciplinary group of researchers, applying a developmentally informed multi-dimensional biopsychosocial lens to pediatric pain research in the clinical and real-world settings.

### Speaker 1 Abstract Title: The influence of parent-child reminiscing about past pain on children’s prosocial development.

**Speaker 1**: Maria Pavlova, MSc, University of Calgary, Department of Psychology, Calgary, AB, Canada. mpavlova@ucalgary.ca @mariavpavlova

**Speaker 1 Abstract**: Parent-child verbal exchanges create a powerful context within which pediatric pain experiences occur. Previous research has demonstrated the robust influence of parent-child verbal interactions on the levels of children’s pain-related distress during immediate painful experiences (e.g., immunizations). To date, no research studies have focused on how parents and children talk about past painful experiences. Overall, parent-child reminiscing about past events shapes multiple aspects of children’s development. Developmental research has demonstrated pronounced between-parent differences in reminiscing styles. Parents who reminisce in an elaborative manner (i.e., use open-ended questions, emotion-laden language, and explanations) have children with more optimal developmental outcomes (e.g., better developed autobiographical memory, language, emotion regulation) as compared to parents who engage in repetitive reminiscing characterized by closed-ended questions, control over the conversation, and repetitions. Our preliminary data revealed significant within-parent differences in reminiscing about past events involving physical (i.e., pain) versus emotional (i.e., sadness) distress. It may be suggestive of different socialization patterns for pain versus sadness. Pavlova will present findings from a new developmental, lab-based study examining how this differential reminiscing about past distressing events is associated with preschooler’s prosocial behaviours (i.e., empathic responses to pain versus sadness in others) and emotion regulation. The potential for language-based interventions and clinical applications will be discussed.

### Speaker 2 Abstract Title: The adverse long-term effects of pain-related stress in the NICU and the role of parents in improving developmental outcomes.

**Speaker 2**: Ruth E. Grunau, PhD, University of British Columbia, Department of Pediatrics, Vancouver, British Colombia, Canada. rgrunau@bcchr.ca

**Speaker 2 Abstract**: Stress from multiple sources such as daily invasive procedures and maternal separation during hospitalization in the neonatal intensive care unit (NICU) contributes to poorer neurobehavioral development of infants born very prematurely (born 2–4 months early). While hospitalized, these fragile neonates are exposed to repetitive pain and stress of routine procedures (approximately 10 per day), at a time of rapid brain development and programming of stress systems. Pain-related stress of invasive procedures in very preterm infants contributes to long-term changes in brain microstructure and functions as well as in stress regulation, leading to poorer cognition and behavior (after accounting for clinical factors associated with prematurity). Parent involvement with their infant in the NICU promotes better brain development, and parenting style after hospital discharge can ameliorate adverse effects of early stress. Evidence for effects of early pain-related stress and how parenting can improve brain development and outcomes will be presented.

### Speaker 3 Abstract Title: Managing infant vaccination-related pain: Is preventing insensitivity better than promoting sensitivity?

**Speaker 3**: Dr. Rebecca Pillai Riddell, PhD, York University, Department of Psychology, Hospital for Sick Children, University of Toronto, Toronto, Ontario, rpr@yorku.ca, @drbeccapr

**Speaker 3 Abstract**: Children undergo multiple needle procedures (e.g., vaccinations) during the first year of life. The needle procedures are often associated with high levels of pain and pain-related fear. Parent behaviours may exacerbate or, on the contrary, alleviate infant pain-related distress. Dr. Pillai Riddell will present a new measure of eight parent behaviours that increase infant distress. The measure has been developed specifically for use in the vaccination setting and provides an innovative, valid, reliable, and feasible method of assessing parent behaviours. Additionally, it provided the opportunity to re-examine sensitivity in the OUCH Cohort (760 infant-parent dyads followed over the first year of life). New analyses suggest that the future of infant pain management in the vaccination context may need to focus more on teaching parents what not to do rather than what to do. While both types of parent behaviours predict infant pain responses, preventing insensitivity may be a more influential way of alleviating infant pain-related distress.

**Learning Objective 1**: To understand and discuss the differences in parent-child reminiscing about past distressing events and their association with children’s prosocial behaviours.

**Learning Objective 2**: To discuss the impact pain-related stress on brain development and the role of parents in reducing detrimental effects of pain on children’s developmental outcomes.

**Learning Objective 3**: To discuss new ways of using the power of parents to manage pediatric pain across medical contexts.

## Temporomandibular Disorders: Insights from Musculature, Brain, and Genes

Barry Sessle, Iacopo Cioffi, Massieh Moayedi 0000-0002-7324-2540, and Shad Smith

**CONTACT** Barry John Sessle barry.sessle@dentistry.utoronto.ca

© 2019 The Author(s). Published with license by Taylor & Francis Group, LLC.

This is an Open Access article distributed under the terms of the Creative Commons Attribution License (http://creativecommons.org/licenses/by/4.0/), which permits unrestricted use, distribution, and reproduction in any medium, provided the original work is properly cited.

**Symposium Chair**: Prof. Barry Sessle MDS, PhD, DSc(h.c.) University of Toronto, Faculty of Dentistry, Toronto (ON), Canada. Barry.sessle@dentistry.utoronto.ca

**Symposium Abstract**: Temporomandibular disorders (TMD) commonly manifest jaw muscle pain and represent the most common chronic orofacial pain disorder. TMD affect about 12% of Canadians and pose a significant socioeconomic burden on society.

Although several risk factors are associated with myofascial TMD (mTMD), clear organic causes for TMD pain have not been proven. This ambiguity contributes to the frequent misdiagnosis and hence mistreatment of mTMD and poses a significant and unnecessary burden on patients and the healthcare system. About 30% of individuals with TMD report pain up to at least 5 years after treatment regardless of the type of management they have received. This relatively high rate of treatment resistance is partly related to uncertainties about the mechanisms underlying mTMD. There is a clear unmet need for clarifying the peripheral and central mechanisms of TMD in order to develop novel treatment strategies. This symposium will present new research findings and discuss novel research modalities that have advanced our understanding of TMD and promise to lead to the development of such treatments. A particular strength of this symposium is the convergence of evidence across different disciplines (genetics, muscle physiopathology, and brain imaging) with regard to orofacial pain mechanisms.

### Speaker 1 Abstract Title: Functional and structural muscular signatures of chronic temporomandibular disorders

**Speaker 1**: Dr. Iacopo Cioffi, DDS, PhD, Faculty of Dentistry, University of Toronto, University of Toronto Centre for The Study of Pain, Toronto (Ontario), Canada. iacopo.cioffi@dentistry.utoronto.ca

**Speaker 1 Abstract**: Temporomandibular disorders (TMD) of muscular origin (mTMD) represent the most common form of TMD, affecting more than 60% of patients with a TMD. There is evidence that an important pathophysiological element of chronic mTMD involves changes in the masticatory muscles, as well as in the peripheral and the central nervous system. Studies have suggested that mTMD is associated with peripheral abnormalities in jaw muscle blood perfusion which could lead to local tissue injuries and peripheral sensitization. Eventually, persistent peripheral nociceptive inputs may induce plastic changes in the pain-related brain circuits, thereby contributing to the transition from an *acute* to a *chronic* painful condition if early resolution does not occur (spontaneously or by treatment).

In this presentation, Dr. Cioffi will discuss recent evidence about pathological mechanisms contributing to chronic myogenous TMD and novel research findings. Using near-infrared spectroscopy, Dr. Cioffi’s team has demonstrated that abnormalities in jaw muscle blood perfusion are already present in healthy individuals at risk for TMD. Furthermore, he will present novel data that indicate that the muscles of mastication of patients with chronic TMD present with structural abnormalities. These muscular signatures provide new diagnostic opportunities for the early detection of TMD, and potential novel therapeutic targets.

### Speaker 2 Abstract Title: Structural and functional brain and trigeminal nerve abnormalities in temporomandibular disorders (TMD)

**Speaker 2**: Dr. Massieh Moayedi, PhD, University of Toronto, Ontario, Canada, m.moayedi@dentistry.utoronto.ca, @massihmoayedi

**Speaker 2 Abstract**: Dr. Moayedi will discuss neural and brain abnormalities in orofacial pain. First, he will discuss brain plasticity driven by an ecological model of persistent orofacial pain in healthy participants. This model will show how 5 days of persistent low-level pain can drive structural and functional changes in the brain. Next, he will present evidence for both peripheral and central neural contributions to idiopathic TMD. Specifically, he will provide an overview of structural and functional brain abnormalities as assessed with magnetic resonance imaging (MRI) in TMD patients, compared to healthy individuals. Finally, he will contextualize these findings based on the results of a systematic review and meta-analysis of brain imaging studies of orofacial pain. Contrasting peripheral and central changes in acute pain and in TMD provides mechanistic information about chronic pain pathophysiology in TMD, and allows for the identification for brain-based biomarker signatures.

### Speaker 3 Abstract Title: Discovery of novel mechanisms for orofacial pain disorders through genome wide approaches.

**Speaker 3**: Dr. Shad Smith, PhD, Center for Translational Pain Medicine, Duke University, Durham, North Carolina, USA. shad.smith@duke.edu

**Speaker 3 Abstract**: TMD is recognized as a complex condition with biological and psychosocial risk factors, including a substantial genetic component. Dr. Smith will present an overview of recent findings of genetic variants associated with painful TMD. Genome-wide and bioinformatics approaches have revealed etiological mechanisms underlying TMD, representing novel pathways of vulnerability. Dr. Smith will present data from stratified analyses that indicate certain pathways may be distinct between sexes. Convergent lines of evidence from genetic, epigenetic, and transcriptomic studies that point to immunological as well as neurological mechanisms will be discussed. He will summarize the state of knowledge about genetic contributors to orofacial pain and its management and explore possible applications for personalized and targeted treatment of TMD.

**Learning Objective 1**: Attendees will improve their understanding of jaw muscle physiopathology

**Learning Objective 2**: Attendees will learn about novel diagnostic approaches for orofacial pain

**Learning Objective 3**: Attendees will be able to identify novel methods for phenotyping orofacial pain

## Pain and the Extracellular Matrix

Laura S. Stone, Lisbet Haglund, Arkady Khoutorsky, and Maral Tajarian

**CONTACT** Laura S. Stone laura.s.stone@mcgill.ca

© 2019 The Author(s). Published with license by Taylor & Francis Group, LLC.

This is an Open Access article distributed under the terms of the Creative Commons Attribution License (http://creativecommons.org/licenses/by/4.0/), which permits unrestricted use, distribution, and reproduction in any medium, provided the original work is properly cited.

**Symposium Chair**: Laura S. Stone, PhD, McGill University, Alan Edwards Centre for Research on Pain, Faculty of Dentistry, Montreal, Quebec, Canada. Laura.s.stone@mcgill.ca; @laurasstone

**Symposium Abstract**: The field of pain research has placed great emphasis on the mechanisms by which neuronal and glial cells regulate pain. In this symposia, we will highlight new insights into the role of the extracellular matrix (ECM) in pain generation and regulation. The workshop will cover mechanisms of pain regulation by the ECM in peripheral tissues (intervertebral discs) as well as in the spinal cord and the brain. The panelists will present data from murine models and from studies with human tissue samples.

### Speaker 1 Abstract Title: Extracellular Matrix Fragments and Toll-like Receptors as drivers of Low Back Pain and Disc Degeneration.

**Speaker 1**: Lisbet Haglund, PhD, Orthopedic Research Lab, McGill Scoliosis and Spine Group, Department of Surgery, McGill University; Shriner’s Hospital, Montreal, QC, Canada, lisbet.haglund@mcgill.ca, @HaglundLisbet

**Speaker 1 Abstract**: Intervertebral disc degeneration is the most common etiology of chronic low back pain. Degeneration is characterized by several changes including a breakdown of the extracellular matrix (ECM) of the Intervertebral Disc (IVD), which is composed of mainly collagen type II and proteoglycans like aggrecan. Furthermore, catabolic proteases, such as matrix metalloproteinases (MMP), and proinflammatory cytokines, increase during degeneration and contribute to ECM breakdown and development of pain. Neurotrophins, such as nerve growth factor (NGF), which is strongly linked to back pain, also increase. The early stages of disc degeneration that lead to these catabolic changes are poorly understood. However, toll-like receptors have recently been suggested to play a role in disc degeneration and pain generation. TLR’s were originally characterized in innate immunity, but is also activated by endogenous ligands found in discs that are termed “alarmins”, such as fragmented hyaluronic acid, aggrecan, and fibronectin. TLR’s are expressed by IVD cells of non-degenerating discs and activation of TLR’s on disc cells increases cytokines, proteases and neurotrophins. Therefore, TLR’s may contribute to the early progression of painful disc degeneration and throughout the course the pathology. In fact, there is strong evidence that chronic TLR inhibition decreases behavioral signs of low back pain, pain-related neuroplasticity and disc inflammation in SPARC-null mice. Therefore, TLRs are potential therapeutic targets to slow disc degeneration and reduce pain.

### Speaker 2 Abstract Title: Remodeling of Spinal Extracellular Matrix Modulates the Development of Pain Hypersensitivity

**Speaker 2**: Arkady Khoutorsky, PhD, DVM, McGill University, Department of Anesthesia, Faculty of Medicine and Dentistry, Montreal Quebec, Canada. arkady.khoutorsky@mcgill.ca

**Speaker 2 Abstract**: In the nervous system, neurons and glial cells are embedded within an extracellular matrix (ECM). In the brain, the ECM restricts synaptic and structural plasticity; thus, enzymatic digestion of ECM enhances acquisition of memories, and promotes cognitive flexibility and extinction. However, it remains unknown whether remodeling of the spinal cord ECM contributes to pathological changes in dorsal horn nociceptive circuits and pain hypersensitivity. Here, we present evidence that the spinal ECM is reorganized following peripheral injury. Transcriptomic data indicate that peripheral nerve injury leads to dramatic changes in the levels of mRNAs encoding components of the ECM in the dorsal horn of the spinal cord. The immunohistochemical analysis revealed that ECM is altered following injury. Remarkably, a digestion of the spinal ECM using chondroitinase ABC (chABC) significantly promoted the development of mechanical hypersensitivity following nerve injury. Collectively, our data indicate that the spinal ECM restricts nociceptive plasticity in the spinal cord. Nerve injury leads to a reorganization of the spinal ECM, contributing to the development of nociceptive hypersensitivity.

### Speaker 3 Abstract Title: The hippocampal extracellular matrix regulates pain and memory dysfunction after peripheral injury

**Speaker 3**: Maral Tajarian, PhD. Queens College, City University of New York, Biology Department, Queens, NY, USA; Maral.tajerian@qc.cuny.edu; @maraltajerian

**Speaker 3 Abstract**: Chronic pain is a heavy burden for the individual and society, presenting with multiple co-morbid psychiatric disorders, including mood alterations and cognitive impairment. These observations have prompted the study of pain-related brain neuroplasticity, mainly focusing on the hippocampus both due to its role in cognition and memory as well as in modulating the overall pain experience. The reported functional, anatomical, and biochemical changes in the hippocampi of chronic pain patients and animal pain models suggest a pivotal role for hippocampal plasticity in the maintenance of pain and implies a level of flexibility in the extracellular environment in which these cells function. This line of investigation has not been pursued to date, both due to the neuro-centric nature of pain research and the technical difficulties of studying extracellular components.

Here we show structural and biochemical alterations in the hippocampal extracellular matrix (ECM) that are linked to pain, cognitive dysfunction, and cellular plasticity in a mouse model of chronic pain. We report deficits in working and location memory that are associated with increased hippocampal LTP, decreased dendritic branching and complexity, altered ECM microarchitecture and decreased ECM rigidity, and changes in the levels of key ECM components. We also report a reduction in specialized ECM nets around inhibitory interneurons, potentially accounting for the increased LTP. These results delineate extracellular mechanisms of pain-related brain plasticity, thereby offering new therapeutic targets that could modulate already-established central nervous system alterations present in chronic pain.

**Learning Objective 1**: Upon completion of this session, attendees will be able to describe the extracellular matrix plasticity that parallels chronic pain in the intervertebral disc, spinal cord, and brain.

**Learning Objective 2**: Upon completion of this session, attendees will be aware of various biophysical and biochemical tools that could be used to study the extracellular matrix in peripheral and central tissues.

**Learning Objective 3**: Upon completion of this session, attendees will demonstrate knowledge in various mechanisms by which the extracellular matrix can be targeted for the treatment of chronic pain.

## Mental Expectations and Neurobiological Determinants of Treatment Outcomes

Javeria Ali Hashmi, Ian Beauprie, A. Vania Apkarian, and Mary E. Lynch

**CONTACT** Javeria Ali Hashmi javeria.hashmi@dal.ca

© 2019 The Author(s). Published with license by Taylor & Francis Group, LLC.

This is an Open Access article distributed under the terms of the Creative Commons Attribution License (http://creativecommons.org/licenses/by/4.0/), which permits unrestricted use, distribution, and reproduction in any medium, provided the original work is properly cited.

**Symposium Chair**: Mary E Lynch, MD FRCPC, Dalhousie University, Department of Anesthesia, Pain Management & Perioperative Medicine, Halifax, Nova Scotia, Canada, mary.lynch@dal.ca

**Symposium Abstract**: It is recognized that endogenous pain relief systems, such as opioid circuitry in the brain, contribute to treatment outcomes of pain. Several neuroimaging studies have consistently highlighted that brain circuits are equipped to adjust pain intensity through learning, motivation and attention systems. Another phenomenon validated in several recent studies is that prior mental states and associated brain activity are significant indicators of intrinsically mediated changes in symptoms that occur on starting a new treatment. Thus, whether an individual has the endogenous capacity to mentally engage and respond to treatment is determined by patterns of brain connectivity. That optimally pre-configured brain circuits are a pre-requisite for better treatment outcomes is a potentially useful observation and needs wider acknowledgement to be clinically useful. New conceptual models and analysis techniques that look at macro-level brain structure and function in large-scale data are quickly revolutionizing this ability. Blue-sky research goals to predict, deploy and enhance these intrinsic responses are seeing a quick surge and may soon change how we diagnose and treat chronic pain. An important implication of these new approaches is that endogenous analgesia and placebo responses will be no longer seen as a non-specific or cryptic response, relevant only to clinical trials and devoid of value in the clinic. This symposium will highlight the role of the brain, the associated mechanisms and the psychological and clinical factors that shape the endogenous aspects of treatment response.

### Speaker 1 Abstract Title: “I am here for my oxy and my medical marijuana—I know it will work!”: clinician perspective on patient expectations in chronic pain clinics.

**Speaker 1**: Ian Beauprie, MD, FRCPC, Dalhousie University, Department of Anesthesia, Pain Management & Perioperative Medicine, Halifax NS, Canada, i.beauprie@dal.ca

**Speaker 1 Abstract**: The majority of adult patients present to pain specialists with fixed or strong expectations. Some come with modality-specific goals as outlined in the deliberately provocative title. The patient focus can be on a treatment not an outcome. The promise of technology such as spinal cord stimulation can evoke miraculous hopes of regaining normal function. Others arrive jaded and pessimistic asking: “what are you going to do when this doesn’t work?” Patients are generally unaware of number-needed-to-treat statistics and the relatively small effect sizes of many treatments. The result of high expectations can be seen in response to therapy: patients can have a honeymoon response to the first hoped-for intervention, then see effect wane over several treatments. If care provided by a clinic differs greatly from the patient expectation, compliance can be low. Those of us who treat patients can be unaware of the cues we provide: white coats, hospital environments, invasive technologies or highly advertised therapeutics. As responsible clinicians we want to deliver the most effective, evidence-based care. Understanding the strong effect of patient expectations on initial therapeutic response can improve buy-in with multidisciplinary care and protect our patients from unwise use of treatments with poor risk/benefit ratio.

### Speaker 2 Abstract Title: Theory, mechanisms and teleological roots of expectation effects on pain therapy

**Speaker 2**: Javeria Ali Hashmi, Bpharmacy, MSc, PhD, Department of Anesthesia, Pain Management & Perioperative Medicine, Dalhousie University, Halifax, Nova Scotia, Canada, Javeria.hashmi@dal.ca, @netphys1

**Speaker 2 Abstract**: Placebo effect is an intriguing but frequently misunderstood phenomenon. Experimental and clinical studies have delved into this phenomenon to demonstrate that the body generates physiological responses that aid the process of treatment. Despite a large number of convergent studies, the debate continues on how to most effectively direct this research into clinically meaningful endpoints. A major barrier for applying this knowledge in therappies is the ambiguity around the term placebo response. In this talk, first the issues with the term placebo response will be discussed to make the assertion that veridical improvements that occur independent of drug action are formed by the contextual factors through a mental cueing process. Acknowledging this fact allows us to comprehend the significance of endogenous systems and advance better conceptual and utilitarian models of how physiological capacity aids therapeutic processes. In addition, teleological role of mental cueing will be explained to postulate how we may have developed this response through evolution and antiquity. The significance of underlying brain mechanisms will be discussed with an emphasis on predictive processes and learning mechanisms that shape the mental cueing response.

### Speaker 3 Abstract Title: Chronic pain as addiction and as an exaggerated memory.

**Speaker 3**: A. Vania Apkarian, PhD, Professor of Physiology, Anesthesia, PM&R, Northwestern University, Feinberg School of Medicine, a-apkarian@northwestern.edu

**Speaker 3 Abstract**: Accumulating evidence shows that chronic pain is dependent on brain emotion and motivation related circuitry, specifically the mesocorticolimbic system. Within this system addiction circuitry and memory circuitry seem to play critical roles in the control of transition from acute to chronic pain. I will also discuss the personality profile of a chronic pain sufferer and its implications regarding therapeutics. Human and rodent model data will be reviewed along these lines and the integration of such diverse mechanisms into a general concept of pain will be discussed.

**Learning Objective 1**: Given the recognized magnitude of the placebo response (expectation effect), should a responsible clinician seek to eliminate it or amplify it?

**Learning Objective 2**: To overview theories and neurobiological mechanisms that mediate expectation effects on pain in experimental and clinical models.

**Learning Objective 3**: To understand that neurobiological and personality factors can predict the development of chronic pain and treatment outcomes.

## Stress and Cognitive Processes Regulating the Experience of Pain and Touch

Robert Bonin, Frank Porreca, and Massieh Moayedi 0000-0002-7324-2540

**CONTACT** Robert P. Bonin rob.bonin@utoronto.ca

© 2019 The Author(s). Published with license by Taylor & Francis Group, LLC.

This is an Open Access article distributed under the terms of the Creative Commons Attribution License (http://creativecommons.org/licenses/by/4.0/), which permits unrestricted use, distribution, and reproduction in any medium, provided the original work is properly cited.

**Symposium Abstract**: Pain is considered to be an “unpleasant sensory and emotional experience”. However, the relative pleasantness or unpleasantness of a sensory experience can be highly variable. The environmental, physiological, and cognitive context can profoundly affect how pain is experienced. For example, stress can both precipitate pain and amplify the unpleasantness of noxious stimuli. The relationship between context and pain has been used in cognitive therapies designed to alleviate or diminish chronic pain.

In this symposium, we will examine the interplay between environmental and cognitive context on the perception and response to noxious and innocuous stimuli. First, Dr. Robert Bonin will discuss new work using optogenetic approaches to study how environmental conditions modulates response to gentle tactile stimuli. He will describe how mouse preference for the activation of sensory afferents responsive to gentle touch is abolished by stress in a manner dependent of the production of corticosterone. Next. Dr. Frank Porreca will describe a potential new mechanism underlying the relationship between stress and pain in functional pain states. His work reveals a kappa-opioid receptor mediated hyperalgesic circuit within the central amygdala that increases descending facilitation. Finally, Dr. Massieh Moayedi will describe data examining the contextual modulation of pain. He will demonstrate how interventions to cognitively re-evaluate the experience or response to a pain stimulus can modulate the perceived unpleasantness and neurophysiological response to pain. Together, these studies provide new mechanistic insight into the relationship between context and the cognitive and physiological response to noxious and innocuous stimuli.

### Speaker 1 Abstract Title: Modulation of responses to gentle touch stimuli by physiological and environmental factors

**Speaker 1**: Robert Bonin, Ph.D. University of Toronto, Leslie Dan Faculty of Pharmacy. Toronto, Ontario, Canada. rob.bonin@utoronto.ca. Twitter: @rpbonin

**Speaker 1 Abstract**: Sensory processing and the perception of noxious and innocuous tactile stimuli is not a fixed process. The perceived aversiveness or pleasantness of tactile stimuli can abruptly change in response to changes in physiological or environmental conditions. This “affective” plasticity can be clearly observed in how our response to gentle stroking of the forearm by another person can feel pleasant or repulsive in different conditions. An abnormal response to socially-relevant physical stimuli may have profound effects on social behaviour, and possibly underlie disorders associated with abnormal social behaviour, such as Autism Spectrum Disorder. However, it is unclear how this rapid sensory plasticity arises or what physiological factors dictate whether identical tactile stimuli are perceived as pleasant or unpleasant.

Using preclinical animal models, we have developed a combination of behavioural and optogenetic approaches to isolate and investigate this form of tactile sensory plasticity. We observed that mice exhibit a preference for gentle physical touch or optogenetic activation of MrgprB4^+^ sensory afferents that respond to gentle touch. However, the preference for optogenetic activation of MrgprB4^+^ afferents was abolished when stimulation was provided in an brightly-lit, aversive environment. We further revealed that the acute modulation of response to MrgprB4^+^ activation is mediated by the stress-related hormone, corticosterone.

Overall, these findings shed light on the mechanisms underlying “affective” sensory plasticity driven by environmental and physiological factors, and may indicate a new approach for reducing the unpleasantness of noxious and innocuous stimuli.

### Speaker 2 Abstract Title: Stress-induced descending facilitation from amygdala kappa opioid receptors in functional pain

**Speaker 2**: Frank Porreca, PhD. University of Arizona, Department of Pharmacology. Tuscon, AZ. USA. frankp@email.arizona.edu.

**Speaker 2 Abstract**: Many patients suffer from chronic pain in the absence of identifiable injury. Such pains are termed “functional” and include irritable bowel syndrome, temporomandibular joint disorder, fibromyalgia, migraine and others. Functional pain patients experience pain free periods that are interrupted by attacks of pain that can persist for variable periods of time. The chronification of these pain disorders has been linked to the number and frequency of attacks suggesting that repeated nociceptive episodes promote and maintain a state of central sensitization. Functional pain patients commonly identify stress as a key trigger of pain episodes but neurobiological mechanisms remain to be determined.

Here, we examine the novel hypothesis that in sensitized states, stress-induced kappa opioid receptor (KOR) signaling in the amygdala promotes functional pain responses. We have developed an injury-free rodent model of stress-related functional pain based on hyperalgesic priming with opioids. Opioids have been shown to produce hyperalgesia in humans and in animals. Following resolution of opioid-induced hyperalgesia (OIH), and in the absence of stress, animals have normal pain responses. This hyperalgesic priming, however, produces a state of latent sensitization marked by the presence of stress-induced hyperalgesia associated with a loss of descending noxious inhibitory controls (DNIC). Using behavioural and electrophysiological approaches, we further show that the loss of DNIC is prevented by blockade of kappa opioid receptor (KOR) signaling within the central nucleus of the amygdala (CeA).

These findings reveal a previously unknown stress-related, KOR-mediated hyperalgesic circuit from CeA that may promote decreased resilience to stress.

### Speaker 3 Abstract Title: The meaning of a painful stimulus modulates neurophysiological responses

**Speaker 3**: Massieh Moayedi, PhD. University of Toronto, Faculty of Dentistry. Toronto, Ontario, Canada. m.moayedi@dentistry.utoronto.ca, Twitter: @massiehmoayedi

**Speaker 3 Abstract**: The experience of pain can be modulated through its perceived meaning. The context in which you experience pain can vastly change the outcome of the experience. I will discuss two studies of contextual modulation of pain. First, we trained a cohort of healthy participants to re-evaluate an experimental pain stimulus through a brief pain-focused cognitive intervention, compared to a cohort that received a non-pain focused intervention. We found that the pain focused intervention significantly and selectively reduced pain unpleasantness, as well as the area of secondary hyperalgesia. This reduction was correlated to pain catastrophizing.

In a second study, we investigated the electrocortical correlates of a withdrawal to a pain stimulus. When they felt a painful laser pulse, participants would release a button (reaction time), and withdraw their hand and press a button close to their body (response time). We found that the N2 wave predicted the withdrawal reaction and response times. We then paired this withdrawal movement to a punishment, every time they would press the button closer to their body, participants would receive an intense electric shock. We found that reframing the context of this withdrawal movement disrupted the relationship with the N2 wave.

Together, these data show that the context of a painful experience can modulate our neurophysiological responses to pain.

**Learning Objective 1**: To understand how optogenetics can be used to investigate acute changes in tactile sensory processing in freely behaving animals.

**Learning Objective 2**: To learn how stress and stress hormones can modulate the central processing of sensory stimuli and the modulation of pain by descending noxious inhibitory control.

**Learning Objective 3**: To gain insights into how cognitive expectations of pain can modulate the experience of pain.

## Prioritizing Pain Provincially: The Need for a Comprehensive Approach

Maria Huspith, Fiona Campbell, Susan Tupper, and John X. Pereira

**CONTACT** Maria Huspith nicki@painbc.ca

© 2019 The Author(s). Published with license by Taylor & Francis Group, LLC.

This is an Open Access article distributed under the terms of the Creative Commons Attribution License (http://creativecommons.org/licenses/by/4.0/), which permits unrestricted use, distribution, and reproduction in any medium, provided the original work is properly cited.

**Symposium Chair**: Maria Huspith, Director, Strategic Initiatives, Pain BC, Vancouver, BC, Canada, nicki@painbc.ca, @painbc

**Symposium Abstract:** Recognizing the efforts and accomplishments in prioritizing pain in provinces and territories across the country, this symposium will explore the development process of three emerging provincial pain strategies.

While the presentations will focus on the Ontario, Saskatchewan, Alberta, and British Columbia pain strategies, we will be speaking to the complexities involved in system transformation efforts. This includes engaging a broad range of stakeholders with different interests and priorities, as well as the interconnected and sometimes competing components within pain strategies. By exploring in detail the efforts underway in these three provinces as a starting point, the symposium will promote discussions on navigating change in other provinces as well as broader system change efforts at a national level.

### Speaker 1 Abstract Title: Ontario Chronic Pain Network *Pain Strategy*; structure, function, achievements

**Speaker 1**: Fiona Campbell, BSc, MD, FRCA, President Canadian Pain Society, Co-director Ontario Chronic Pain Network (Pediatric), Hospital for Sick Children, Toronto, Ontario, Canada, fiona.campbell@sickkids.ca, @DrFCampbell

**Speaker 1 Abstract:** Introduction: The *Ontario* Chronic Pain Network is a partnership between the MOHLTC, providers working in chronic pain programs, and patients. There are pediatric and adult subnetworks, with integrated activities and overlap in committee structure. History, structure and function of the network will be described with emphasis on recent innovations. Methods: The network was established in 2013, to improve awareness of chronic pain, and address gaps in access to care, education and research. An Advisory Board (Program Directors from each site, representation from MOHLTC, subcommittee chairs, patients) provides oversight, leadership and strategic advice to the network and subcommittees (Research & Evaluation, Education, Clinical Care, Strategic Development). Results: In 2014, MOHLTC provided $20M in base funding for 5 pediatric & 13 adult hospitals, and 1 community clinic, leading to: i) **Improved access to care**: increased capacity in clinics, reduced wait times, streamlined referral and intake processes, ii) **Program development**: programs hired interprofessional teams to deliver high quality care, creation of first Canadian intensive inpatient & day treatment program for paediatric pain, and iii) **Research & Education**: piloted a common registry in an adult and pediatric hospital, organized 3 annual pediatric education days, Project ECHOs have linked 864 providers and over 320 organizations. Conclusions and Next Steps: Having improved access to care, developed standards, increased research capacity and education outreach, the pediatric and adult networks will collaborate to address system gaps and build capacity in high priority areas: transition from paediatric to adult care, people in remote and rural communities, and Indigenous populations.

### Speaker 2 Abstract Title: SaskPain: Saskatchewan’s journey to develop a provincial pain strategy

**Speaker 2**: Susan Tupper, PT, PhD, Saskatchewan Health Authority, Pain Strategy, Saskatoon, Saskatchewan, Canada, susan.tupper@usask.ca, @smtpt

**Speaker 2 Abstract:** Introduction: The Saskatchewan Pain Society (SaskPain) is a newly incorporated non-profit society with a mission to promote better understanding of pain and advocate for pain services throughout Saskatchewan (SK). Findings from two stakeholder dialogues and two online surveys that inform ongoing work to develop a provincial pain strategy will be described. Methods: Facilitated dialogues were held in 2014 and 2016 with key stakeholders from across the province. Narrative responses were collated and summarized into strategy themes by members of the planning committee with expertise in qualitative data analysis. Online surveys conducted in 2014 and 2018 contributed to identification of priorities for SaskPain. Results: Facilitated dialogues in 2014 (n = 143) and 2016 (n = 42) were summarized into two reports and distributed for feedback to clinicians, academic institutions, healthcare regulatory bodies, and contacts at the SK Ministry of Health. The following primary drivers of change were identified: knowledgeable and engaged healthcare providers, knowledgeable and engaged public, specialty services for pain assessment and management, and infrastructure to support best-practice and change at the microsystem, organizational and environmental levels. Change strategies were categorized under the following 4 pillars for future work: provincial pain foundation, pain education reform, regional pain management practice/quality improvement, and research/knowledge translation. Surveys in 2014 (n = 83) and 2018 (n = 25; preliminary data) confirmed priorities for program development and focus for each of the SaskPain working groups. Conclusions: Using broad stakeholder consultation, actionable strategies have been identified to develop a foundation of knowledge, resources and advocacy to support accessible, coordinated pain management services in Saskatchewan.

### Speaker 3 Abstract Title: Alberta Pain Strategy: A Truly Provincial Collaboration

**Speaker 3**: John X. Pereira, MD CM CCFP CIME CEDIR VI, President, Pain Society of Alberta, Co-Chair, Alberta Pain Strategy, Calgary, Alberta, Canada, john@johncpc.com, @Alberta_Pain (Twitter handle for the entire PSA)

**Speaker 3 Abstract:** Introduction: The Alberta Pain Strategy is a collaboration of Alberta Health Services (AHS), the Pain Society of Alberta (PSA), the University of Calgary, the University of Alberta, and over a dozen other stakeholder groups. More than twenty patient and family representatives contributed to the strategy. It builds on the previous PSA 2015 Provincial Pain Strategy document. Methods: Facilitated dialogues were held in 2017 and 2018 with key stakeholders from across the province meeting in Jasper, Red Deer, Banff and via videoconferencing. An engagement survey was sent province-wide in September of 2018. Guided by a steering committee, work was done by an AHS planning team and the strategy was divided into three working groups: Acute Pain, Chronic Pain and Opioid Use. Results: The engagement survey (N = 171) showed significant agreement with the proposed vision statement of “Achieving excellence in pain management across the lifespan for all Albertans” and the priorities of the three working groups. The Opioid Use working group generated debate and a wide variety of opinions. A draft of the new five-year Alberta Pain Strategy (2018–2023) will be presented in Banff in October 2018. Conclusion: Work on the new Alberta Pain Strategy has moved rapidly from its launch just one year ago. A team with dedicated work hours for this endeavour has been essential.

**Learning Objective 1**: To learn about different system transformation approaches in the development of emerging provincial pain strategies

**Learning Objective 2**: To understand the objectives and the different components within provincial pain strategies and the relationship among them

**Learning Objective 3**: To situate research and practice in the broader policy context to help improve the lives of people living with pain

## Opioid De-Prescribing: Practical Advice from a Patient, a Pharmacist and a Physician on How to Safely and Successfully Taper Opioids

Andrea Furlan 0000-0001-6138-8510, Kirk Foat, Laura Murphy 0000-0002-4787-8879, and Andrew Smith

**CONTACT** Andrea Furlan andrea.furlan@uhn.ca 550 University Avenue, 7-141, Toronto Rehab, UHN, Toronto ON Canada M5G 2A2

© 2019 The Author(s). Published with license by Taylor & Francis Group, LLC.

This is an Open Access article distributed under the terms of the Creative Commons Attribution-NonCommercial License (http://creativecommons.org/licenses/by-nc/4.0/), which permits unrestricted non-commercial use, distribution, and reproduction in any medium, provided the original work is properly cited.

**Symposium Chair**: Andrea Furlan, MD PhD, University of Toronto, Department of Medicine, Toronto, ON, Canada, andrea.furlan@uhn.ca twitter @adfurlan

**Symposium Abstract**: Canada is the second largest prescriber of opioids in the world. The number of opioid prescriptions in Canada increased 6.8% between 2012 and 2016, from 20.2 million in 2012 to 21.5 million in 2016. The most common opioids prescribed in Canada include strong opioids such as hydromorphone, fentanyl, and oxycodone. Long-term and high dose opioids are associated with significant risks, which include death, opioid poisoning, sleep apnea, hypogonadism, depression and opioid-induced hyperalgesia. The population with the highest of opioid use also has the highest risks from opioids; more than 20% of seniors received at least one prescription of opioid in 2015–16. (CIHI, 2018) The 2017 Canadian Opioid Guideline recommends that “for patients with chronic noncancer pain who are currently using 90 mg morphine equivalents of opioids per day or more, they taper opioids to the lowest effective dose, potentially including discontinuation, rather than making no change in opioid therapy”. De-prescribing opioids can be challenging to both clinicians and patients. The challenges are related to selecting the proper patients, choosing the best tapering regimen and sticking to the plan. The most common barriers from a clinician’s perspective include: lack of knowledge, skills, resources, motivation and empathy. Patients also lack knowledge about the reasons why tapering is necessary, and they fear worsening of pain and withdrawal symptoms. There is an urgent need to educate both clinicians and patients about appropriate tapering of opioids with the goals of improving quality of life of patients with chronic pain.

### Speaker 1 Abstract Title: Getting to Zero

**Speaker 1**: Kirk Foat, BA Sociology University of Western Ontario and patient expert in two research studies related to opioids., kirkfoat@gmail.com twitter @KirkFoat

**Speaker 1 Abstract**: Kirk Foat survived a harrowing accident only to realize that his work had only begun. After a radical radial flap procedure that saved his hand from a life threatening leptospirosis infection in 2008 he was prescribed opiates to help with the wide ranging pain he was managing. He overcame PTSD only to realize a few years later that the opiates that assisted with his ability to do the physio required to regain hand function were negatively impacting his quality of life. Listen to Kirk detail the alternatives sought to manage pain and his self initiated taper plan and how he was able to get to zero. He’ll detail from a patient perspective what being a high dose opiate user for chronic pain was like, and what he learned tapering and what life is like without opiates managing pain. Kirk Foat and Dr. Andrea Furlan both appeared in the CBC radio show *White Coat Black Art* originally aired on November 3, 2017 (“How one man got off prescription opioids and got his life back”).

### Speaker 2 Abstract Title: Guidance on opioid tapering in the context of chronic pain: Evidence, practical advice and frequently asked questions

**Speaker 2**: Laura Murphy, PharmD, Toronto Rehabilitation Institute- University Health Network, Pharmacy, Toronto, ON, Laura.Murphy@uhn.ca

**Speaker 2 Abstract**: The 2017 Canadian Guideline for Opioids for Chronic Non-Cancer Pain suggests opioid tapering should be considered for adults with chronic noncancer pain on ≥90 mg morphine equivalent dose daily. There is limited evidence available to guide the design of tapering regimens. This speaker is a pharmacist with extensive clinical experience in opioid tapering. She will provide practical guidance to inform the individualization of opioid-tapering regimens based on the collective experience of a group of expert pharmacists across Canada. The Guideline directs prescribers to collaborate with pharmacists to support opioid tapering; a multidisciplinary or team-based approach has been associated with increased success. Dr. Murphy will outline a motivational approach that pharmacists and other team members can use with patients to initiate and monitor an opioid taper to increase the success of opioid reduction while minimizing adverse events and harm.

### Speaker 3 Abstract Title: Challenging cases of opioid tapering.

**Speaker 3**: Andrew Smith, MDCM, Centre for Addiction and Mental Health, Toronto, ON, Canada, Andrew.Smith@CAMH.ca twitter @AJKSmithMD

**Speaker 3 Abstract** (250 words or less): The 2017 Canadian Guideline for Opioids for Chronic Non-Cancer Pain suggests that patients who are using opioids and experiencing serious challenges in tapering be referred to a multidisciplinary pain program. This speaker is a pain and addiction medicine specialist who leads an interprofessional team whose mandate is to treat patients with complex chronic pain along with aberrant drug-related behviours, possible substance use disorders and/or mental health co-morbidities. Dr. Smith will explore the concepts of dependence on prescription opioids, chemical coping, and opioid use disorder – which exist on a spectrum of prescription opioid use, the role of buprenorphine to assist patients in challenging tapering situations, and the evidence to support its use in managing chronic pain.

**Learning Objectives**: At the end of this symposium participants will be able to identify appropriate patients for tapering opioids, use evidence-based protocols for opioid tapering, and to use empathy and compassion when helping patients to achieve the lowest possible dose of opioids to help them manage their chronic noncancer pain.

## Getting Your Message Across: Learning to Communicate about Pain with Different Stakeholders and Knowledge Users

Christine Chambers 0000-0002-7138-916X, Neil Andrews, Maria Hudspith, and Erica Ehm

**CONTACT** Christine Chambers christine.chambers@dal.ca IWK Health Centre, 5850/5980 University Avenue, Halifax, NS, B3K 6R8, CANADA

© 2019 The Author(s). Published with license by Taylor & Francis Group, LLC.

This is an Open Access article distributed under the terms of the Creative Commons Attribution License (http://creativecommons.org/licenses/by/4.0/), which permits unrestricted use, distribution, and reproduction in any medium, provided the original work is properly cited.

**Symposium Chair**: Dr. Christine Chambers, PhD, Departments of Pediatrics and Psychology & Neuroscience, Dalhousie University, Halifax, NS, Canada, Christine.chambers@dal.ca, @DrCChambers

**Symposium Abstract**: Being able to effectively communicate about pain with different types of stakeholders and knowledge users (e.g., patients, caregivers, policy makers, the public at large) is critical in order to improve health outcomes and quality of care for patients with pain. Yet most of the formal training pain researchers and clinicians receive prepares them only for communicating with other researchers and clinicians. This workshop will provide an overview of effective communication strategies for different types of stakeholders and knowledge users, and will capitalize on the expertise and experiences of three professional communicators. Neil Andrews, Executive Editor of Pain Research Forum/RELIEF, will present on strategies for making science more accessible to the public. Maria Hudspith, Executive Director of Pain BC, will present on strategies to communicate effectively with policy makers to promote change. Erica Ehm, founder of YMC.ca (an award winning on-line publication for Canadian mothers) and owner of Ehm & Co (a digital agency specializing in the mother market) will talk about strategies to effectively communicate and engage with Canadian parents. In addition to sharing effective strategies, common mistakes and pitfalls will also be discussed. We will use Twitter during the symposium to take polls, share information, and promote engagement. The symposium will conclude with an interactive question and answer period. The role of effective communication in promoting dissemination and implementation of evidence to change practice and improve pain for patients in pain will be emphasized.

### Speaker 1 Abstract Title: Making Science Accessible to the Public: What a RELIEF!

**Speaker 1**: Neil Andrews, MS, MA, Pain Research Forum, International Association for the Study of Pain (IASP), 1510 H St. N.W., Suite 600, Washington, D.C., USA, neil@painresearchforum.org,@NeilAndrews, @PainResForum)

**Speaker 1 Abstract**: In January 2016, the IASP Pain Research Forum (PRF), an interactive online community of pain researchers, launched a companion web site called RELIEF (relief.news). RELIEF is a freely available and editorially independent news web site for patients and the wider public that translates the research PRF covers into accessible language and ideas. Its mission is to provide everyone touched by or interested in the problem of chronic pain with knowledge and information about the latest research, in order to raise awareness of and elevate public discourse about chronic pain, rally support for research, and speed progress towards new treatments. It’s a lofty ambition – but translating complicated science for non-specialists is not an easy task. This presentation will describe RELIEF’s efforts to do just that, focusing on the experience we have gained in developing plain language summaries of research news, feature articles, interviews and podcasts for our readers, with a discussion of the challenges we have faced and the opportunities ahead of us. A particular focus will be the PRF Correspondents program, a science communications training program that helps early-career pain researchers develop their ability to communicate science to the public on venues like RELIEF, as well as to their fellow pain investigators on PRF. This talk will describe lessons learned from the Correspondents program thus far, and how our experience training young scientists to become better writers and reporters can be used to improve communication of science to the public.

### Speaker 2 Abstract Title: Getting Pain on the Agenda: Communicating with Policy Makers to Catalyze Change

**Speaker 2**: Maria Hudspith, MA (Educational Studies), Executive Director, Pain BC, 320– 1508 West Broadway, Vancouver, BC, Canada, maria@painbc.ca, @PainBC

**Speaker 2 Abstract**: Pain BC, a collaborative non-profit organization, has been using a “collective impact” model to drive policy change in BC. Working with people in pain, clinicians, researchers, business leaders, government and other non-profits, Pain BC has gotten chronic pain on the agenda in our province. This has resulted in a comprehensive provincial pain strategy and has motivated health care organizations and academic institutions to take action on pain through their own policies and programs. It has also mobilized people with lived experience who are active in all aspects of Pain BC’s work – from the conception of initiatives to delivery of programs, to advocacy and governance. This model offers a blueprint for a Canadian pain strategy and is informing emerging policy work at the federal level. Pain has been undertreated and under recognized in the health care system. It has also long been ignored by decision makers – from local politicians to health authority and hospital CEOs to provincial, national and international policy makers. How can researchers, clinicians and patient advocates communicate with policy makers and bring about real and impactful change for people who live with pain? How can policy levers be used to transform the way pain is understood and treated? What strategies are effective in engaging policy makers in taking action on pain? This talk will describe the “how to” influencing policy with the PainBC strategy as one of the demonstrated wins of the process.

### Speaker 3 Abstract Title: The Art Behind the Science: Communicating and Engaging with Parents

**Speaker 3**: Erica Ehm, YMC.ca and Ehm & Co, 33 Chaplin Crescent, Toronto, Ontario, Canada, erica@yummymummyclub.ca. @EricaEhm

**Speaker 3 Abstract**: Many pain researchers and clinicians try to reach and engage with parents, but have no formal training or experience in doing so. Considered a “voice of her generation”, Erica Ehm rose to fame in the 80’s as host and cultural curator at Much Music, Canada’s national music channel. She is also the founder of the award winning niche platform, YMC.ca, and digital agency Ehm & Co., both focused on the desirable parent market. Erica is considered a pioneer in the digital marketing and publishing arenas, with expertise in creating compelling integrated programs for mothers and building meaningful relationships with parents over social media. In this presentation, Erica will share cutting-edge communication strategies for engaging with parents, including mothers and fathers. She will share insights from marketing on current and evolving ways to engage with Canadian parents that pain researchers and clinicians could consider adopting in their own work. Erica will also share lessons learned in her role as the media partner in the award-winning CIHR funded #ItDoesntHaveToHurt initiative, the goal of which was to educate parents about children’s pain management more quickly and creatively than through traditional means. At over 150 million impressions (i.e., content views) to date, #ItDoesntHaveToHurt is the most successful campaign that Ehm & Co has ever run. Erica will share her insights on how she was able to help spark and fuel an ongoing conversation about pediatric pain research amongst Canadian parents.

**Learning Objective 1**: To learn of the challenges facing efforts to translate complicated science into understandable language and how to overcome them.

**Learning Objective 2**: To understand the collective impact model as it applies to advancing policy change and to learn strategies for communicating with provincial and national policy makers

**Learning Objective 3**: To gain understanding of cutting-edge strategies from marketing that could be used to communicate and engage with parents about pain research and management.

## Time for a PEP Talk: Building the Evidence for Patient Engagement in Pain

Carley Ouellette, Dawn Richards, Christine Chambers 0000-0002-7138-916X, and Kathryn Birnie 0000-0002-8223-8834

**CONTACT** Kathryn Birnie kathryn.birnie@sickkids.ca

© 2019 The Author(s). Published with license by Taylor & Francis Group, LLC.

This is an Open Access article distributed under the terms of the Creative Commons Attribution License (http://creativecommons.org/licenses/by/4.0/), which permits unrestricted use, distribution, and reproduction in any medium, provided the original work is properly cited.

**Symposium Chair**: Carley Ouellette, BScN RN, McMaster University, Nursing, Hamilton, Ontario, Canada; ouellc1@mcmaster.ca; @carleyouellette

**Symposium Abstract**: Public or patient engagement in research is “…research being carried out ‘with’ or ‘by’ members of the public rather than ‘to’, ‘about’, or ‘for’ them” (INVOLVE, 2018). Patient engagement represents a shift from the traditional view of patients as research participants to one that empowers patients, otherwise identified as “people with lived experience”, as partners and co-builders on research teams. Evidence suggests that engaging patients as collaborators enhances the quality, appropriateness, and relevance across stages of the research process. This includes increased study enrolment and decreased attrition, improved data collection tools, more effective dissemination and implementation of study findings, better researcher-community rapport, and closer alignment of research objectives to patient-identified priorities. However, challenges to greater uptake of patient engagement identified by researchers include difficulties identifying representative and appropriate patients, uncertainty about the scope of patients’ roles, perceived lack of evidence regarding the impact of patient engagement, and the need for researcher education and culture change as a prerequisite. Thus, there is a need for continual knowledge generation and reflective practice regarding patient engagement in health research. The objective of this symposium is to illustrate diverse, meaningful, and active partnership of people with lived experience with pain and their families, in pain research governance, priority setting, research conduct, and knowledge translation. This symposium draws from multiple expert perspectives, including two individuals with lived experience with pain and extensive involvement with patient engagement (symposium chair and first speaker), as well as two researchers leading national patient engagement practice in pain research (speakers).

### Speaker 1 Abstract Title: Integration of Lived Experience throughout the SPOR Chronic Pain Network

**Speaker 1**: Dawn Richards, PhD, Chronic Pain Network, McMaster University, Hamilton, Ontario, Canada, dawn.p.richards@gmail.com, @TO_dpr

**Speaker 1 Abstract**: People living with chronic pain bring a unique perspective to research. Living with a condition 24 hours a day, 7 days a week, provides a perspective of urgency, relevancy, and resilience. The SPOR Chronic Pain Network (CPN) has harnessed the capacity, passion, and perspective of individuals living with chronic pain throughout its governance, operations, and research activities. These community efforts began prior to CPN’s funding by CIHR: in parallel with its grant application efforts, extensive work was undertaken to uncover research priorities for those living with chronic pain. CPN now has engaged approximately 30 partners with lived experience who play roles in its governance, committee membership, research projects, and in helping lead patient engagement and related training efforts (with ebb and flow to accommodate for health and life reasons). With so few similar models to draw from in the research space, CPN’s efforts have been based on guidance available in the literature (published and grey), best practice examples, involving people who have experience in patient engagement in other areas, good intentions and trial and error. Experiences will be shared from the CPN about the initial approach to integrate the lived experience, continued efforts throughout its operations to date, and insights that continue to evolve as a result of evaluating and examining current patient engagement efforts. The presentation will be delivered by an individual whose insights in to patient engagement and research are due to her own lived experience with pain and training as a basic scientist.

### Speaker 2 Abstract Title: Patient engagement lessons learned from #ItDoesntHaveToHurt and #KidsCancerPain social media initiatives

**Speaker 2**: Christine Chambers, PhD, Department of Pediatrics and Psychology & Neuroscience, Dalhousie University, Halifax, NS, christine.chambers@dal.ca, @DrCChambers

**Speaker 2 Abstract**: The failure to meaningfully and actively engage patients in health research and care has been identified as a barrier to uptake of research findings and improvements in care delivery. While the arguments for engaging patients in research are compelling (e.g., improved research quality and relevance, reduced research waste etc.), there is limited empirical research available to guide its practice or theoretical basis. Using two successful social media initiatives as case studies, this session will evaluate the experience of patient as research partners. Dr. Christine Chambers will provide an overview of the #ItDoesntHaveToHurt and #KidsCancerPain social media initiatives and the role that patient partners had in guiding project design, implementation, and evaluation. Dr. Chambers will present data from 14 patient partners (93% parents, 67% mothers, 56% aged 40–49) who completed a structured interview at the end of each initiative. Survey results indicated that parents felt listened to (100%), that meetings respected their schedule (83%), and that they were able to share their personal experience as a patient as part of the team (94%). On a scale from 1 to 7, patient reported feeling comfortable with the project (M = 6.22, SD = .81) and that their comments impacted project decision making (M = 5.76, SD = 1.56). While common lessons learned emerged in both groups (e.g., the power of partnership and collaboration), differences emerged in suggestions for improvement, which will be explored in this presentation. This investigation offers novel empirical data on the value of engaging patient partners in pain research.

### Speaker 3 Abstract Title: #PartneringForPain: Empowering the patient and parent voice to co-build the future of pediatric chronic pain research

**Speaker 3**: Kathryn Birnie, PhD; Lawrence S. Bloomberg Faculty of Nursing, University of Toronto & Child Health Evaluative Sciences, The Hospital for Sick Children, Toronto, Ontario, Canada; kathryn.birnie@sickkids.ca; @katebirnie

**Speaker 3 Abstract**: This presentation will outline co-produced activities from a Canadian patient engagement project involving people with lived experience with pediatric pain, parents, researchers, healthcare providers, and advocacy groups. Patient and parent partner input and expertise are sought throughout project activities from design to dissemination.

Outcomes include (1) a patient engagement registry linking patients/families with researchers to partner on research teams; and (2) a top 10 list of research priorities identified by patients, families, and healthcare providers using an internationally established partnership priority setting process (James Lind Alliance). First steps of the #PartneringForPain priority setting process are completed, including a national survey with 215 respondents from across Canada: 40% people with lived experience with pediatric chronic pain, 26% family members, and 34% healthcare providers. From the over 530 ideas submitted, priorities related to health systems issues (e.g., access and standardization of care, transition to adult services,), school (e.g., education for personnel, accommodations), healthcare providers’ beliefs and behaviours, public and healthcare provider education and awareness, social and peer support, chronic pain etiology, assessment/diagnosis, and prognosis, as well as specific treatment modalities (e.g., cannabis). Ongoing next steps include an online interim priority setting survey and final priority setting workshop (held in November 2018). The final top 10 patient- and parent-identified research priorities will be presented. This talk will offer learning from our own experience brokering and managing patient-researcher partnerships, as well as identifying benefits and challenges still to be addressed. Quotes from patient and parent partners about their experience will be shared.

**Learning Objective 1**: To understand and critically view how patient partners’ roles were established and are evolving in a national research network.

**Learning Objective 2**: To understand the experience of parent partners involved in social media initiatives and ways to improve it in the future.

**Learning Objective 3**: To see meaningful integration of patient and parent partners as members of a research team, and empowerment of patient and parent voices to identify pain research priorities.

## New Directions in Chronic Pain: What Might the Future Hold?

Jordi Perez, Martin Koltzenburg, and Patrick Mantyh

**CONTACT** Jordi Perez jordi.perez@muhc Alan Edwards Pain Management Unit, McGill University Health Centre, 1650 Cedar Ave, Montreal, Quebec, Canada H3G1A4

© 2019 The Author(s). Published with license by Taylor & Francis Group, LLC.

This is an Open Access article distributed under the terms of the Creative Commons Attribution License (http://creativecommons.org/licenses/by/4.0/), which permits unrestricted use, distribution, and reproduction in any medium, provided the original work is properly cited.

**Symposium Chair**: Jordi Perez, MD, PhD, FIPP.

Associate Professor, Anesthesia and Director, Cancer Pain Fellowship, McGill University, Montreal, QC Canada. Associate Medical Director, Alan Edwards Pain Management Unit. Director, MUHC Cancer Pain Program

**Symposium Abstract**: Among adults in Canada, approximately 15–19% experience chronic noncancer pain – defined as a painful condition that persists for three months or longer. For most people with this type of chronic pain, it lasts much longer than three months: more than half of adults in Canada with chronic pain report suffering with it for more than 10 years. Two of the most prevalent types of chronic pain – and most common causes of disability in Canada – are low back pain and osteoarthritis, affecting up to 22% and 14% of Canadian adults, respectively. The burden of chronic pain weighs heavily on patients and society, with direct and indirect costs greater than that of cancer, heart disease, and HIV combined. Against this backdrop, in this symposium we will explore the challenges clinicians face in the pharmacological management of chronic pain and review our evolving understanding of the pathophysiology of chronic pain. Building on this science, we will discuss the mechanism of action, analgesic properties, efficacy, and safety of potential new treatments that modulate nerve growth factor (NGF) in chronic pain pathways, as well as the possible clinical applications of anti-NGFs to improve patient outcomes.

### Speaker 1 Abstract Title: Unmet Needs and Challenges in Chronic Pain

**Speaker 1**: Jordi Perez, MD, PhD, FIPP. Associate Profe-ssor, Anesthesia and Director, Cancer Pain Fellowship, McGill University, Montreal, QC Canada. Associate Medical Director, Alan Edwards Pain Management Unit. Director, MUHC Cancer Pain Program

**Speaker 1 Abstract**: Chronic pain presents a significant burden on patients’ lives: patients have reported that pain interferes significantly with various aspects of their daily living, including normal work, walking ability, and recreational and social activities. In addition to non-pharmacological therapies for chronic pain (self-management, physical activity, psychological therapies), current recommended non-opioid and opioid pharmacological treatments have demonstrated at least limited efficacy to improve chronic pain and help patients regain function, though not necessarily to premorbid levels. The continuing and increasing burden on the healthcare systems and the economy, however, demonstrates not only the complexity of chronic pain, but also the limitations of current treatment options; in Canada, the estimated medical costs to treat low back pain alone ranges from $6–12 billion dollars annually. Efficacy of medications is often moderated by significant side-effect profiles, from gastrointestinal disturbances to opioid addiction and even death. In this presentation, attendees will review current data about the effectiveness of currently recommended pharmacological options to treat chronic pain, including low back pain and osteoarthritis, as well as safety and tolerability. Participants will also have an opportunity to compare their own clinical challenges in chronic pain management with those identified in the literature, including treatment adherence, side effects management, and abuse/misuse potential.

### Speaker 2 Abstract Title: The Mechanisms of Chronic Pain: What We Know in 2019

**Speaker 2**: Martin Koltzenburg, MD, Dr. med. Professor and Chair, Clinical Neurophysiology, University College London, London, UK. Neurologist and Head of the Department of Clinical Neurophysiology, The National Hospital for Neurology and Neurosurgery at Queen Square. Phone: 011 44 20 3448 4752. m.koltzenburg@nhs.net

**Speaker 2 Abstract**: Pharmacological treatment options for chronic pain have not significantly altered over the past two decades. While there are studies and guidelines that can help guide the optimal selection of medication for specific chronic pain conditions, there remains a clinical gap in providing significant relief for many patients with chronic pain, including low back pain and osteoarthritis. In this presentation, we will review the possible reasons for this gap by delving deeper into our current understanding of the pathways and underlying mechanisms of chronic pain, and how existing pharmacologic treatments – both non-opioid and opioid – modulate these pathways and mechanisms. Building on this knowledge, participants will consider advances in the understanding of pain pathophysiology, including nerve growth factor (NGF), and the potential for mechanism-based treatments for chronic pain.

### Speaker 3 Abstract Title: Advancing Knowledge in Chronic Pain Management: The Role of Nerve Growth Factor

**Speaker 3**: Patrick Mantyh, PhD, JD. Professor of Pharmacology, Department of Pharmacology, University of Arizona, Tucson, AZ, USA. Phone: (520) 626–0742. pmantyh@email.arizona.edu

**Speaker 3 Abstract**: A greater understanding of nerve growth factor (NGF) in the pathophysiology of chronic pain has provided opportunities to identify and investigate novel treatments. Pharmaco-logical agents that target the NGF pathway (anti-NGFs) and its effect on pain initiation and maintenance are under investigation, including fasinumab and tanezumab. In this presentation, participants will gain an understanding of the mechanism of action of anti-NGFs in chronic pain conditions, including osteoarthritis and low back pain. Results of efficacy and safety studies of anti-NGFs will be discussed, which will allow participants to consider the potential clinical use of these drugs to help improve functional outcomes and quality of life for patients with chronic pain conditions.

**Learning Objective 1**: Identify unmet needs and challenges in the optimal treatment of chronic pain, including low back pain and osteoarthritis

**Learning Objective 2**: Explain the current understanding of pain pathways and mechanisms and how they are modulated by current pharmacological treatment options

**Learning Objective 3**: Describe the mechanism of action of anti-nerve growth factor drugs in the management of chronic pain and their potential application in the clinical setting

